# Indicator Properties of Baltic Zooplankton for Classification of Environmental Status within Marine Strategy Framework Directive

**DOI:** 10.1371/journal.pone.0158326

**Published:** 2016-07-13

**Authors:** Elena Gorokhova, Maiju Lehtiniemi, Lutz Postel, Gunta Rubene, Callis Amid, Jurate Lesutiene, Laura Uusitalo, Solvita Strake, Natalja Demereckiene

**Affiliations:** 1 Department of Environmental Science and Analytical Chemistry, Stockholm University, Stockholm, Sweden; 2 Finnish Environment Institute, Marine Research Center, Helsinki, Finland; 3 Leibniz Institute for Baltic Sea Research, Rostock-Warnemünde, Rostock, Germany; 4 Department of Fish Resources Research, Institute of Food Safety, Animal Health and Environment, Riga, Latvia; 5 Open Access Centre for Marine Research, Klaipėda University, Klaipeda, Lithuania; 6 Latvian Institute of Aquatic Ecology, Riga, Latvia; 7 Lithuanian Environmental Protection Agency, Klaipėda, Lithuania; CNRS, FRANCE

## Abstract

The European Marine Strategy Framework Directive requires the EU Member States to estimate the level of anthropogenic impacts on their marine systems using 11 Descriptors. Assessing food web response to altered habitats is addressed by Descriptor 4 and its indicators, which are being developed for regional seas. However, the development of simple foodweb indicators able to assess the health of ecologically diverse, spatially variable and complex interactions is challenging. Zooplankton is a key element in marine foodwebs and thus comprise an important part of overall ecosystem health. Here, we review work on zooplankton indicator development using long-term data sets across the Baltic Sea and report the main findings. A suite of zooplankton community metrics were evaluated as putative ecological indicators that track community state in relation to Good Environmental Status (GES) criteria with regard to eutrophication and fish feeding conditions in the Baltic Sea. On the basis of an operational definition of GES, we propose mean body mass of zooplankton in the community in combination with zooplankton stock measured as either abundance or biomass to be applicable as an integrated indicator that could be used within the Descriptor 4 in the Baltic Sea. These metrics performed best in predicting zooplankton being in-GES when considering all datasets evaluated. However, some other metrics, such as copepod biomass, the contribution of copepods to the total zooplankton biomass or biomass-based Cladocera: Copepoda ratio, were equally reliable or even superior in certain basin-specific assessments. Our evaluation suggests that in several basins of the Baltic Sea, zooplankton communities currently appear to be out-of-GES, being comprised by smaller zooplankters and having lower total abundance or biomass compared to the communities during the reference conditions; however, the changes in the taxonomic structure underlying these trends vary widely across the sea basins due to the estuarine character of the Baltic Sea.

## Introduction

Assessing community response to altering habitats is of great theoretical and practical importance if we are to understand anthropogenic impacts on aquatic ecosystems and recommend adequate management strategies. Although environmental indicators are always simplifications and snapshots of interacting ecological processes, a jointly monitored set of indicators characterizing community structure and functionality can facilitate assessment of ecosystem state [1]. In the Baltic Sea Action Plan (BSAP), the Contracting Parties to the Helsinki Convention agreed to evaluate periodically whether the targets of the Action Plan are met by using indicator-based assessments [2]. A year after the BSAP, the EU Marine Strategy Framework Directive (MSFD) reiterated the need for the protection, sustainable management and restoration of the European seas [3]. MSFD is the first directive that requires a systematic assessment of the environmental status of all European regional seas. In particular, the directive specified assessment requirements, listed common pressures on marine ecosystems, and defined qualitative descriptors for the good environmental status (GES) of the marine environment. Although each Member State has the responsibility to define specific GES objectives, the MSFD requires that monitoring methodologies must be compatible within and between regional seas and consistent with ongoing monitoring programs at a regional and international level. Also, the MSFD specifically requests the monitoring of phytoplankton and zooplankton for the descriptor *Food Webs* (Descriptor 4) and emphasizes the need for indicator approach. In this context, development of ‘top-down’ and ‘bottom-up’ indicator metrics at the regional level is a pre-requisite for assessment of trophic conditions in marine ecosystems.

In aquatic ecosystems, a hierarchical response across trophic levels is commonly observed, with higher trophic levels showing a more delayed or a weaker response to environmental stressors affecting food web functioning than lower trophic levels [4]. Therefore, alterations in planktonic primary producers and primary consumers are considered the most sensitive ecosystem responses to anthropogenic stress, including eutrophication [5,6]. Changes in primary productivity due to eutrophication and warming and the consequent reorganization of zooplankton communities have been documented worldwide [7,8]. As shown for temperate lakes, zooplankton taxa often differ in their preferences for the trophic state [9–12]. Moreover, they are of different value as prey for zooplanktivores, because of the taxa-specific variations in size, escape response, and biochemical composition. In the Baltic Sea, alterations in fish stocks and regime shifts received a particular attention as driving forces behind changes in zooplankton [13,14]. With the position that zooplankton has in the food web—sandwiched between phytoplankton and fish (i.e., between eutrophication and overfishing)–understanding of zooplankton responses are a prerequisite for the ecosystem approach to management. Thus, there is a firm recognition of zooplankton role in regional and global biogeochemical fluxes and cycles, mediating transport and balance of particulate and dissolved matter in aquatic systems [15]. However, despite their potential as indicators of environmental changes influencing food web functioning, the use of zooplankton assemblages as indicators of ecosystem state has been limited so far. To date, indicator-based approaches have mostly been developed for freshwater ecosystems [16,17], although applications in coastal areas [18], including the Baltic Sea [19] also exist.

Neglecting zooplankton as a relevant quality element for the assessment of ecological status within EU Water Framework Directive (WFD) has been criticized [7,8,20]. For the development of food web indicators within Descriptor 4, particularly relevant are changes in food-web structure and functioning. With respect to fish feeding conditions, higher absolute or relative abundances of zooplankters with certain body size are usually associated with good food availability [21–23]. Further, increased total zooplankton stocks, due to small-sized plankters [24] with a concomitant decrease in mean zooplankter size [11,19,25], have been associated with eutrophication-driven alterations in the food web structure. Also, the contribution of small-bodied forms increases in concert with increasing frequency and magnitude of cyanobacteria blooms, which is considered as a sign of eutrophication [26,27].

Indicator development requires regional calibration exercises and revision of existing data for responsiveness of zooplankton metrics to relevant pressures [8,20]. Here, we present results of such exercise for long-term zooplankton data originating from different areas of the Baltic Sea. In this exercise, we explored properties of various zooplankton-based metrics derived from the community analysis within HELCOM-guided monitoring as indicators for fish feeding conditions and eutrophication-driven food web changes to gather support for the development of zooplankton indicators within MSFD in the Baltic Sea. In this evaluation, we have not attempted to determine processes that account for the changes in zooplankton in this system, but to establish whether these changes have coincided with local changes in eutrophication status and fish nutritional status. The evaluated indicators should be seen as an early outcome of this work, presenting frames for further indicator development and implementation.

## Materials and Methods

### Zooplankton data and sampling areas

**Fig 1:**
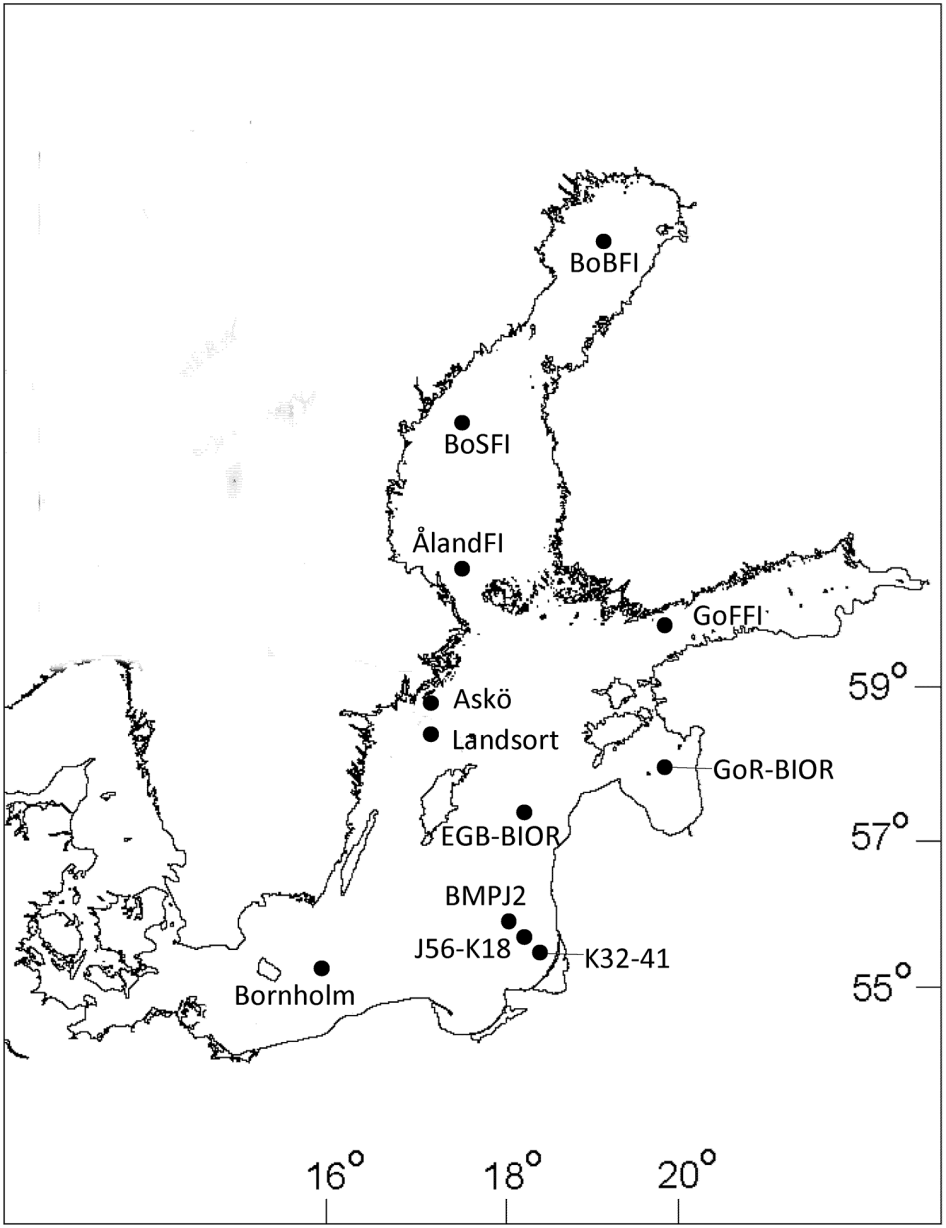
Map of the Baltic Sea indicating sampling sites for zooplankton datasets used in this study. Each dataset is represented by a single circle; when several stations contributed to a dataset, the circle shows the approximate middle of the sampled area. See Table 1 for description of sampling sites and sampling frequencies.

**Fig 2:**
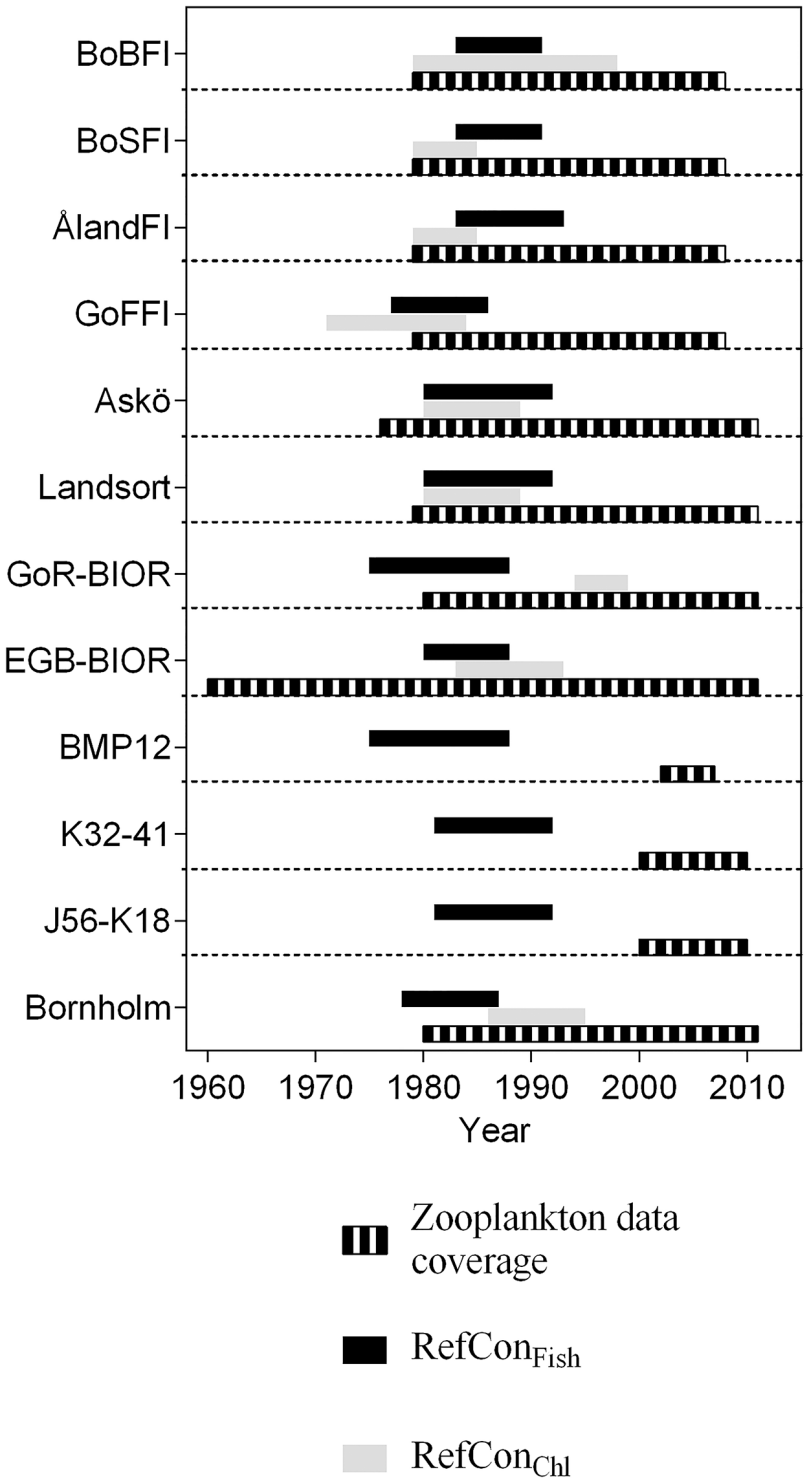
Time coverage for zooplankton data and reference periods based on the existing EQR for Chlorophyll *a* (RefCon_Chl_) and fish body condition (RefCon_Fish_) for each dataset. See Table 1 for details.

The data originate from national Finnish, German, Latvian, Lithuanian and Swedish monitoring programs in the Baltic Sea (Table 1; Fig 1); all data are publicly available from several databases (Swedish Meteorological and Hydrological Institute, SHARK database: www.smhi.se; Baltic Sea mesozooplankton dataset: http://kodu.ut.ee/~riina82/; Data Center of the German Maritime and Hydrographic Agency: http://www.bsh.de/en/, and COPEPOD: http://www.st.nmfs.noaa.gov/copepod/). Sampling locations represent good geographic coverage for coastal and open sea areas in the Gulf of Bothnia, Northern, Central, Southeastern and Southern Baltic proper, and Gulfs of Finland and Riga. Due to considerable variation in sampling frequency between the monitoring programs, the data are restricted to the average values observed during the summer period (June-September) as the most represented in all datasets. This is also the period of the highest plankton productivity as well as predation pressure on zooplankton (S1 Fig) [28–30]. The length of the time series used in this analysis varied from 6 to 51 years (Fig 2).

**Table 1:** Details for the data sets provided by national monitoring programs for indicator testing; deviations in sampling methods from the HELCOM guidelines (i.e., WP2, 100-μm mesh size) are indicated. See S1 Table for details.

Data set code	Country	Area	Stations, geographic coordinates, maximal sampling depth	Time period (gaps)	Sampling frequency^a^	Deviations in sampling methods from the guidelines
Askö	Sweden	Northern Baltic proper	*B1*: 58°48'N 17°37'E; 40 m	1976–2011 (1990, 1993)	8–10	Water bottle^b^ (1983–1988), otherwise WP2, 90-μm mesh size^c^; no flow meter
Landsort Deep	Sweden	Northern Baltic proper	*BY31*: 58°40'N 18°18'E; 459 m	1979–2011 (1981, 1997, 2004–2006)	2–10	WP2, 90-μm mesh size^c^; no flow meter
GoFFI	Finland	Gulf of Finland	*LL7*: 59°30'N 24°30'E; 95 m	1979–2008 (1999)	1^d^	none
GoFFI	Finland	Gulf of Finland	*LL3A*: 60°02'N 26°48'E; 60 m	1979–2008 (1989, 1990, 1999, 2000)	1^d^	none
ÅlandFI	Finland	Åland Sea	*F64*: 60°06'N E 19°05'E, 280 m	1979–2008 (1988–1990, 1997, 1999)	1^d^	none
BoSFI	Finland	Bothnian Sea	*SR5*: 61°02'N 19°20'E; 125 m	1979–2008 (1989, 1997, 1999)	1^d^	none
BoSFI	Finland	Bothnian Sea	*US5B*, 62°21'N 19°34'E; 116 m	1980–2008 (1989, 1997, 1999)	1^d^	none
BoBFI	Finland	Bay of Bothnia	*BO3*^e^: 64°10'N 22°12'E; 100 m	1979–2010 (1989, 1990, 1997–1999)	1^d^	none
BoBFI	Finland	Bay of Bothnia	*F2*^f^: 65°13'N 23°16'E; 90 m	1979–2008 (1983, 1989, 1990, 1997–2000)	1^d^	none
GoR-BIOR	Latvia	Gulf of Riga	23 stations: 57°03'N 23°34'E to 58°15'N 23°01'E; 14 to 57 m	1980–2011	11–31^a^	Juday net, 160 μm mesh size, no flow meter
EGB-BIOR	Latvia	Eastern Gotland Basin	31 stations: 54°54'N 19°15'E to 59°31'N 21°40'E; 25 to 120 m	1960–2011 (1968, 1969, 1973, 1974, 1992, 1993)	3–43^a^	Juday net, 160 μm mesh size, no flow meter
K32-41	Lithuania	Southeastern Baltic proper	4 stations: 55°18'N 20°57'E to 56°01'N 21°01'E; 12 to 15 m	2000–2010	2–4^d^^,^^g^	WP2, 108 μm mesh size (2000–2005), Apstein net, 100 μm mesh size (2009–2010)
J56-K18	Lithuania	Southeastern Baltic proper	6 stations: 55°31'N 20°33'E to 56°01'N 20°50'E; 25 to 62 m	2000–2010	3–6^d^^,^^g^	WP2, 108 μm mesh size (2000–2005), Apstein net, 100 μm mesh size (2009–2010)
BMP12	Lithuania	Southeastern Baltic proper	56°01'N 19°08'E; 120 m	2000–2007	1^d^	WP2, 108 μm mesh size
Bornholm	Germany	Bornholm Sea	*BMPK2*, 55°15'N 15°58'E; 91 m	1980–2011	1^d^	TSK flow meter since 2005, no flow meter before that

^a^if not specified otherwise, this frequency is a number of samples collected during June-September;

^b^23-L water bottle was used to sample water column every 5 m (bottom to surface) and pooled for counting using a 90-μm sieve;

^c^WP2 nets with mesh size of 90 and 100 μm were compared in 2003 in the northern Baltic proper and found to provide statistically similar sampling efficiencies for all relevant zooplankton groups (Gorokhova, unpubl.);

^d^August;

^e^or stations BO3N and/or BO3S located in a close proximity;

^f^or station F2A located in a close proximity;

^g^total for all stations

### The indicators

A set of putative indicators (Table 2) was selected based on existing literature and discussions within Zooplankton Expert Network (ZEN) supported by HELCOM and pelagic biodiversity group (HELCOM CORESET 1). The following metrics of zooplankton communities were evaluated as indicators of change in fish feeding conditions and food web properties caused by eutrophication.

**Table 2:** Indicator description, calculation principles, and rationale. Only species/groups that are included consistently in the zooplankton analysis were used for calculations.

Indicator, units	Parameters used for calculation	Rationale
Total zooplankton abundance (TZA); ind.× 10^3^ m^-3^	Zooplankton^a^ number in the field samples and corresponding volume of water filtered through the net	High zooplankton abundance is primarily related to eutrophication, with rotifers and cladocerans contributing most to the responses.
Total zooplankton biomass (TZB); mg WW m^-3^	Total zooplankton^a^ abundance and individual weights	As above. High biomass of zooplankton may also imply high food availability for zooplanktivorous fish.
Copepod biomass (CB); mg WW m^-3^	Copepod abundance and individual weights	High biomass of large-bodied copepods has been associated with high individual growth in zooplanktivorous fish.
Contribution of copepod biomass to total zooplankton biomass (CB%); %	Copepod abundance, individual weights and total zooplankton biomass	High contribution of copepod biomass has been associated with high individual growth in zooplanktivorous fish.
Microphagous mesozooplankton biomass (MMB); mg WW m^-3^	Microphagous zooplankton^b^ abundance and individual weights	Eutrophication favors small-sized phytoplankton, which in turn favors microphagous filtrators.
Contribution of microphagous mesozooplankton biomass to total zooplankton biomass (MMB%); %	Microphagous zooplankton abundance, individual weights and total zooplankton biomass	As above; the same rationale holds true for the contribution of MMB to total zooplankton.
Mean zooplankter size (MeanSize); μg WW ind^-1^	Total zooplankton abundance and total zooplankton biomass	Microphagous filtrators are most commonly represented by small-sized organisms. They are also negatively selected by zooplanktivorous fish.
Ratio between biomass of cladocerans and biomass of copepods (Cla/Cop)	Cladoceran^a^ biomass and calanoid copepod biomass	Cladocerans are parthenogenic, mostly microphagous filtrators; favoured by eutrophied conditions and bloom-like increases in primary production.
Ratio between biomass of rotifers and cladocerans and biomass of copepods, (RotCla/Cop)	Biomass of cladocerans^a^ and rotifers and biomass of calanoid copepods (all species and stages).	Rotifers are parthenogenic microphagous filtrators; favoured by eutrophied conditions and bloom-like increases in primary production

^a^predators (e.g. *Cercopagis*, *Bythotrephes*, and *Leptodora*) are excluded from these calculations;

^b^tintinnids, rotifers, appendicularians, small (<2 mm) ctenophores, herbivorous cladocerans, pelagic harpacticoids are included in these calculations.

#### Total zooplankton abundance and biomass (TZA and TZB)

In lakes and estuaries, herbivorous zooplankton stocks have been reported to correlate with chlorophyll *a* and phytoplankton biomass at various scales [4,25,31–35], but also with total phosphorus [25]. Total zooplankton stocks often increase with eutrophication, usually as a result of a rise in small herbivores [4,25,36]. Therefore, both TZA and TZB have been recommended as ‘bottom-up’ indicators [8]. Moreover, in coastal areas of the northern Baltic Sea, recruitment of coastal fish was best explained by total zooplankton abundance [37].

#### Copepod biomass, absolute and relative (CB and CB%)

In most areas of the Baltic Sea, copepods are important prey for zooplanktivorous fish, such as sprat and young herring, and fish body condition and weight-at-age (WAA) have been reported to correlate positively to abundance or biomass of copepods [23,38]. Baltic copepods are mostly herbivorous; therefore, this indicator would be indirectly impacted by eutrophication via changes in primary productivity and phytoplankton composition [39]. Direct effects on CB and CB% are expected mostly from predation, although locally, both positive and negative responses can result from climatic changes and natural fluctuations in thermal regime and salinity.

#### Microphagous mesozooplankton biomass, absolute and relative (MMB and MMB%)

Eutrophication favors small-sized phytoplankton, bacterioplankton, and detritus production, thus, promoting microbially-driven energy pathways in the food web [40]. These food resources are particularly accessible for microphagous filtrators: rotifers, herbivorous cladocerans, naupliar stages of copepods and larvaceans. Climatic changes, i.e., increasing temperature and decreasing salinity, are also suggested to promote microbial pathways in the Baltic Sea [41].

#### Mean zooplankter size (MeanSize)

Numerous ecological processes, e.g., growth and metabolic rates, prey size range [42,43] and predator preference for prey [44] are functions of body size. Hence, a shift in zooplankton body size can affect main ecosystem properties—water clarity, rates of nutrient regeneration, and fish abundances [42,43,45,46]. Thus, body size can provide the basis describing functional and structural food web models [46]. Although, the decrease in average zooplankter size can be caused by a variety of factors, such as increased temperature [45,47], eutrophication [48,49], fish predation [47,48,50,51], non-indigenous species introductions [52], and pollution [45], the resulting change implies a community that is well adapted to eutrophic conditions and provides a poor food base for fish. Indeed, as eutrophication progresses, large species are commonly replaced by smaller ones [36], which are also less vulnerable to predation by planktivorous fish [53]. Zooplankton size has been proposed as an index of predator-prey balance, with mean size decreasing as the abundance of zooplanktivorous fish increased and increasing when the abundance of piscivores increased due to trophic cascades [50].

#### Biomass ratios of cladocerans to copepods and of rotifers and cladocerans to copepods (community ratios, Cla/Cop and RotCla/Cop)

In the Baltic Sea, rotifers and cladocerans are important, particularly in summer. In coastal areas with low copepod abundance, cladocerans may become a primary food source for various planktivorous fishes and invertebrate predators [54,55]. Via parthenogenic reproduction, both rotifers and cladocerans can rapidly increase their abundance in favorable conditions. This ability makes them well adapted to the opportunistic use of seasonally changing resources, but also to eutrophication-driven changes in primary productivity. These are also the microphagous taxa, feeding on small-sized algae and, to some extent, on bacteria [56]; therefore, they were included in the calculations of the MMB values. The ratio between cladocerans and calanoid copepods was found to be a good predictor of nutrient enrichment (the Laurentian Great Lakes: [16,57]; Lake Biwa: [4]). Similarly, both relative and absolute biomass of rotifers increased with the trophic state (southern Baltic: [40]; Estonian lakes: [58]; Lake Biwa: [4]) and with chlorophyll concentration (northern Baltic Sea: [19,59]; North-American lakes: [25]). Small cladocerans, such as *Bosmina*, were also reported to respond positively to cyanobacteria blooms, a common sign of eutrophication [26,60]. To calculate these community ratios, we used only herbivorous cladocerans, excluding predatory onychopods (*Cercopagis*, *Leptodora*, and *Bythotrephes*), ctenophores and mysids. These indicators were expected to be directly and positively affected by eutrophication via changes in primary productivity and phytoplankton composition. However, some negative effects might occur due to predation, and both positive and negative effects can result from species-specific responses to climatic changes and fluctuations in thermal regime and salinity.

### Approaches for defining reference periods and boundaries

A fundamental difficulty when using indicators is setting reference conditions. The reference condition can be based on existing reference areas or populations that are in a pristine state, historical records that date back to the time when anthropogenic pressures are considered as being low/absent, or a modeling using related variables with known reference condition to derive the reference state for the variable in question [61]. The main difficulty is the lack of sites that are not currently affected by human activities and data that date back to such reference periods; this holds true also for Baltic zooplankton. Therefore, it would not be feasible to follow this approach for establishing a reference condition for zooplankton as well as for many other ecological groups in the Baltic Sea.

Alternatively, a period within existing time series can be selected to define a reference state when the food web structure was not measurably affected by eutrophication or representing good fish feeding conditions. To define the reference conditions, existing GES for eutrophication-related variables and fish stocks may be applied. This, however, is complicated by the occurrence of sudden changes in the structure and function of the food web, i.e. regime shifts, that have been identified in the Baltic Sea system, including zooplankton [13,62], although we know little about proximate causes and timing of such shifts from areas other than the Central Baltic Sea. Some of these abrupt changes have been linked to eutrophication and fishing, whereas others were related to the climatic and hydrographic conditions. Since the GES boundaries should be in line with the prevailing physiographic conditions and climate, the existence of time periods with different stable states should be acknowledged in the selection of a reference period.

We evaluated two alternative strategies for setting reference conditions for the indicators tested. *The first approach* was to use a long-term average for an entire dataset and corresponding variance and to evaluate deviations from the variability boundaries; this approach is particularly relevant if the time series are very short. *The second approach* was based on basin-specific reference conditions for (1) chlorophyll a concentrations (RefCon_Chl_) representing eutrophication state with no measurable effects on grazers in the food web, and (2) fish feeding conditions (RefCon_Fish_) representing food web structure supporting adequate nutrition for zooplanktivorous fish. To define RefCon_Chl_, we used existing assessment for eutrophication in the sub-basins of the Baltic Sea [63,64]. To define RefCon_Fish_, we used data on growth and stocks of young herring and sprat to identify periods of good feeding conditions for zooplanktivorous fish in the relevant ICES subdivisions. Herring and sprat are dominant species both in the commercial fishery and as zooplanktivores in the Baltic Sea, playing a crucial role in the food web functioning across the sea basins [13,14] and depending on zooplankton availability, particularly during summer (S1 Fig). Therefore, any impact on food availability and population recruitment of these species would affect the ecosystem performance.

### Detection of changes in time-series of indicators using control charts

The principles of process control are well-established in the area of production and operations management [65]. Process control makes use of control charts to determine if the underlying distribution of a measurable variable is undergoing a shift. A control chart uses information about the process variation to examine if the process is moving beyond the expected stochastic variability stated as desirable tolerance limits. If the process is *in control*, then subsequent observations lie within the limits. The hypothesis that the process is *in control* is rejected if the observations fall outside the limits. As a test statistic, control charts employ the controlling mean (*μ*) and specify control limits of *n* × standard deviations (*σ*) above and below the mean or the confidence intervals (CI). The baseline (or reference) conditions are represented by *μ* that can be defined for a selected period or the entire dataset. The time series of the selected metrics of zooplankton community structure were analyzed with combined Shewhart and cumulative sum (CuSum) control charts using SixSigma module in STATISTICA 8.0 (StatSoft, USA). The Shewhart control chart provides enhanced detection of sudden deviations, whereas CuSum methods detect persistent small changes in observed processes or periods when the long-term mean changes [65]. The control charts have been recommended as a tool to interpret environmental monitoring data and to detect abnormal deviations in time series [66,67], including fish [68,69] and zooplankton [70,71].

A factor to consider when interpreting control charts is the control limit values, which is a function of the variability of the data, and, thus, reflects the statistical power to detect a deviation from the baseline. In this study, the upper and lower control limits (UCL and LCL, respectively), were defined as either 99%-CIs around the mean values (for *μ* based on an entire dataset), or using a conservative approach of ±3σ and ±5σ for Shewhart and CuSum control limits, respectively (for *μ* based on either RefCon_Fish_ or RefCon_Chl_) [67–69]. The determination of whether an indicator was beyond the expected limits was carried out over the evaluation period for each data set. Most of the datasets with >12 years of observation tested with Kolmogorov-Smirnov normality test were found to deviate significantly from the normal distribution; particularly, MMB and the community ratios. Therefore, the indicator values were Box-Cox transformed, and all downstream analyses were carried out on the transformed data; all *z*-scores were normally distributed (*p* > 0.2 in all cases). Missing values were predicted by Eigen-Vector Filtering [72].

For each indicator and dataset, once a controlling mean (*μ*_*i*_) and standard deviation (*σ*_*i*_) have been specified based on the chosen baseline period, indicator values (*x*_*i*,*t*_) within the time-series were standardized to *z*-scores (*z*_*i*,*t*_) as:

$$z_{i,t}=\frac{x_{i}{}_{,t}-\mu_{i}}{\sigma_{i}}$$ (1)

As standardized values, *z*-scores enable direct comparison of changes for different sites and variables, irrespective of their absolute values.

To implement our two approaches for setting reference conditions, we specified the *μ*_*i*_ and *σ*_*i*_ of the underlying normal distribution parameters for constructing the control charts. In the first approach, we used all data available (i.e., all years of the monitoring period, including the most recent year). In the second approach, a window of the data corresponding to the selected reference period (Fig 2) representing:

1. RefCon_Chl_ that was defined using a period with environmental quality ratio (EQR) >0.67 and historical data on chlorophyll-a [64,65]; this indicates in-GES state in the system, and
2. RefCon_Fish_ that was set using periods of successful foraging in the relevant ICES subdivisions, when both fish growth assessed as weight-at-age, WAA, or other condition indices; [23,73], and stocks were relatively high [14,74]. Recently, Ljunggren et al. [37] suggested that WAA could be used as a proxy for food availability to relate feeding conditions to fish recruitment in coastal areas of the northern and central Baltic Sea.

To investigate trends in accumulated changes for each indicator in question, a decision-interval CuSum (DI-CuSum) was calculated by recursively accumulating positive and negative deviations separately with two statistics:

$$S_{i}^{+}=\text{max}\left[0,S_{i-1}^{+}+z_{i}-k\right]$$ (2)

for positive deviations (one-sided upper CuSum), and

$$S_{i}^{-}=\text{min}\left[0,S_{i-1}^{-}+z_{i}+k\right]$$ (3)

for negative deviations (one-sided lower CuSum), with *S*_*i = 0*_ = 0 [74]. This scheme is particularly suitable for indicators showing either a positive or negative response and has, for example, been applied for analysis of cod stocks in the North Sea [69]. The *k* value is the allowance value in the process expressed in *z* units of the mean shift one wishes to detect, i.e., deviations smaller than *k* are ignored. The default choice of *k* = 0.5 was applied here, which is considered appropriate for detecting a 1-*σ* shift in the process mean [75].

### Detecting monotonous trends and shifts in zooplankton community structure

For the analysis of trends and sudden shifts, we used *z*-scores calculated for the entire data period. For each indicator, the non-parametric Mann—Kendall test for a monotonic downward or upward trend was applied. Chronological clustering was used to identify homogenous time intervals for zooplankton community structure in each dataset. Chronological clustering produces groups of sequential years, defined by connectedness and a clustering sensitivity parameter *α*. Identification of breakpoints is usually investigated using different α values for the same connectedness level [76]. To detect sudden shifts in our indicator time series, we used *z*-scores calculated for the entire data period and the Euclidean distance function to calculate the dis(similarity) between years using software package Brodgar (www.brodgar.com). The main breakpoints in the time series were calculated using *α* values 0.01, 0.05 and 0.1 and a constant connectedness level set at 50%.

### Logistic regressions linking indicators to the reference periods

Standard binomial regression (logistic regression) was used to evaluate a binary response variable (1 when *in-control* vs. 0 when *out-of-control* years) for RefCon_Fish_ and RefCon_Chl_ as a function of the indicators, utilizing a minimum number of variables. The indicator values were calculated using a baseline for the entire observation period (i.e., using the first approach). The reference and the out-of-control years were selected based on the indicator behavior with RefCon_Fish_ and RefCon_Chl_ baselines (i.e., using the second approach), so that if any of the indicators exceed CuSum control limits for a given year and a given type of the reference state, it was assigned as an *out-of-control* year. Thus, these regression models described the probability of falling inside the RefCon_Fish_ or RefCon_Chl_. In other words, we investigated what indicators were the most informative for predicting whether zooplankton community is within GES. As some indicators would be correlating due to the nature of their calculation, Pearson correlation analysis were used for *z*-scores (entire data period) to evaluate possible redundancy of the indicators as predictors. When selecting predictors, multicollinearity was explored using the correlation analysis results as well as regression diagnostics and Variance Inflation Factor (VIF scores; [77]). In the final models, none of the VIF were ≥ 3, which is well below the cut-off point of 10; thus, the models have not been degraded by collinearity. To identify whether for neighboring areas have similar behavior of the indicators in relation to RefCon_Fish_ or RefCon_Chl_, we included *dataset* as a categorical variable in the regressions. When *dataset* was found non-significant, the resulting regressions were defined as applicable for more than one area. The scaled deviance and the Pearson χ^2^ were used to evaluate the model fit, and the overall best model was determined using Akaike's Information Criteria (AIC). The AIC selects models with high likelihood while penalizing for additional parameters, such that the best model has the smallest AIC. When a more complicated model was not significantly different (p > 0.05) than a simpler model with a similar AIC value, the simpler model was chosen as the best model. Percentages of correct classification cases and odds ratio were used to access prediction accuracy; the classification cut-off was set to 0.5.

## Results

### Variability of the indicator values

**Fig 3:**
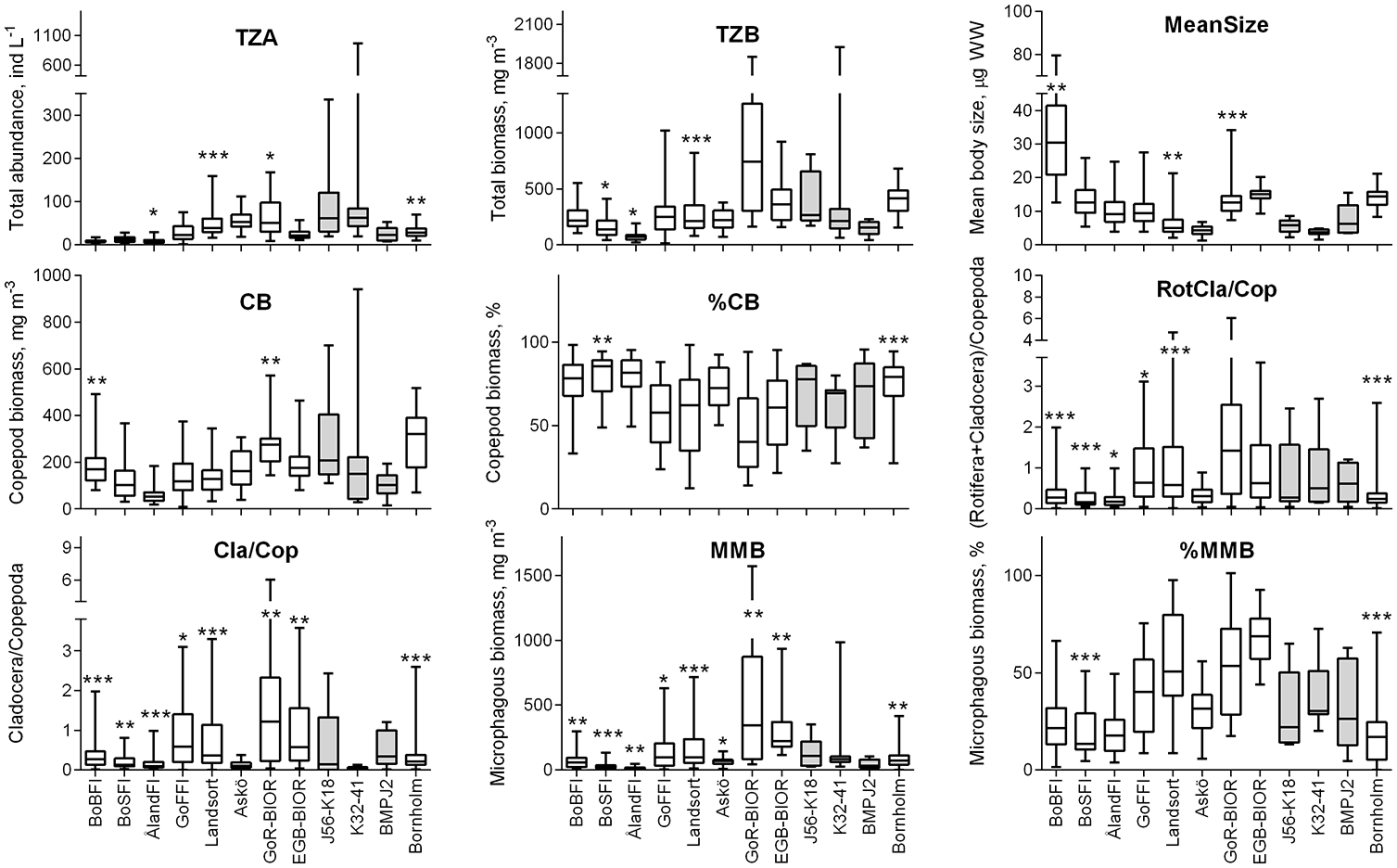
Variability of zooplankton indicators in the analyzed datasets. The datasets are indicated as *Station*. Box-and-whiskers show median, 25 and 75% percentiles, min and max values. Asterisks (*: p< 0.05, **: p < 0.01, and ***: p < 0.001) indicate significant deviations from Gaussian distribution using the Kolmogorov-Smirnov statistics applied to the data sets with ≥18 years of observations. Shaded columns indicate datasets that are <12 years and thus not eligible for normality testing. See Tables 1 and 3 for the details on the indicators and datasets.

For all indicators, there was considerable variability across datasets (Fig 3), particularly for Cla/Cop and MMB. The observed median values for these indicators (non-transformed) spanned 64- and 41-fold range, respectively, with the highest values recorded in the BIOR datasets and the lowest in ÅlandFI and K32-41 datasets. The least variable median values were the percentages (CB% and MMB%), with maximal differences of 3- and 5-fold respectively) and CB, for which a ~5-fold difference was observed between the highest (BIOR) and the lowest (ÅlandFI) values.

The Box-Cox transformation significantly decreased CV% values in most of the indicators (Wilcoxon signed rank test, p < 0.015; S2 Fig). For the transformed data, the highest variability (CV%>50%) was found for the ratios (RotCla/Cop and Cla/Cop), particularly in the short datasets (J56-K18 and K32-41), whereas the lowest CV% values (13–34%) were observed for TZA, MeanSize and MMB, particularly in the relatively long datasets (i.e., Landsort, BIOR and Bornholm; CV% < 20%). For most indicators, the CVs% estimated for the RefCon_Chl_ and RefCon_Fish_ periods were less variable, with MeanSize (<26% in all datasets) and TZB (<32% in all datasets) having the lowest variability (S2 Fig).

### Control charts for indicators with baselines estimated for the entire data sets

#### TZA and TZB

In all datasets, TZA and TZB occasionally exceeded both UCL and LCL (Table 3; S3A and S3B Fig). During the 1960–1980s, low TZA values with out-of-control periods of >5 years were detected in the Gulf of Bothnia and Åland Sea (BoBFI, BoSFI, and ÅlandFI) as well as in the offshore areas of the Baltic proper (EGB-BIOR and Landsort). These periods were followed by generally high TZA in 1990–2000s, with values often exceeding the CuSum-UCL in the same datasets. As a result, significant increasing trends for TZA (BoSFI, ÅlandFI and EGB-BIOR) and TZB (BoSFI and EGB-BIOR) were observed for the entire period (S3A Fig). The opposite TZA and TZB trends occurred in the Gulf of Riga (GoR-BIOR), with a significant overall decrease in both variables. A significant TZB decrease was also found for GoFFI, Askö, and Bornholm (S3A and S3B Fig). Moreover, periods of low out-of-control TZA and TZB values were observed for GoFFI and BIOR data around the middle of the last decade, although these deviations were less pronounced than in the 1980s (S3A and S3B Fig).

**Table 3:** Summary of CuSum analysis (S3 Fig) for all indicators and datasets with >12 years of observations. Periods when UCL or LCL (bold) were violated for >3 consecutive years are shown; the first two digits are omitted for simplicity.

Datasets	TZA	TZB	CB	CB%	MMB	MMB%	RotCla/Cop	Cla/Cop	MeanSize
Entire data period									
BoBFI	**83–92**; 94–03						**85–89**		
BoSFI	**84–90**; 00-		92-9c6						
ÅlandFI	**81–86**			82–87;	**80–87**	**82–87**	**82–87**		83–92; **05-**
GoFFI	93, 96;	**03–08**		**91–95**	**03-**	89–95; 05-	90–96; **05-**	89–96; 05-	89–93
Landsort	**83–91**; 92–98	91–98		**92–96**	92–97	92–96	91–98	91–97	88–96; **00–05**
Askö			88–96; **00-**	85–96;		**84–96**; 98-	**84–96**; 00-	86–93; 98–02	88–95; **97-**
GoR-BIOR	82–86, 88–93		80–88; **01–06**		**03–07**				
EGB-BIOR	**64–73**; 91–95	**61–87**	**63–73**	**90–94**	**62–69**; **95–00**	90–94; **94–00**		61–69; 89–94	**-87**, **96–00**
Bornholm	**04-**	**05-**	**01-**	**00–04**	**91–99**; 00–04	**91–96**; 00–09	00–08	01–05	**00–04**, **06–10**
RefCon_Fish_									
BoBFI	95-								**00-**
BoSFI	03–08								
ÅlandFI									**03-**
GoFFI				05-	**05-**	**03-**	**05-**	**99-**	**99-**
Landsort						**07-**			**98-**
Askö		**98-**	**97-**	**96-**		96-	96-	96-	**96-**
GoR-BIOR	**95-**	**96-**	**97-**	00-	**95-**	**94-**	**99-**	**99-**	
EGB-BIOR	**-65**	**-66**	**-66**		**96–02**				**96–01**, **06-**
Bornholm	**04-**	**96-**	**97-**	**96-**	**91–02**; 00–04	**91–98**; 00-	99-	98-	**98-**
RefCon_Chl_									
BoBFI						**99–03**			
BoSFI	99-	94-	01-			**98–06**			
ÅlandFI	88-			**90-**	87-	93–97, 02-	88-	86-	**90-**
GoFFI		**04-**		06-	**03-**	**99-**	**05-**	01-	**94-**
Landsort	92-								**97-**
Askö		**98-**	**97-**	**99-**		**97-**	96-	93-	**96-**
GoR-BIOR	-89		-86		-87	-87			
EGB-BIOR	**-70**	**-64**, **97–07**			**-64**, **96-**	**96-**	**98-**	**01-**	
Bornholm	**04–08**	**04-**	**00-**	**01-**	02-	00-	00-	98-	**97-**

#### CB and CB%

During the 1990s, a shift from high to low CB and CB%, with values exceeding the LCL, was observed for Askö and Bornholm datasets, which resulted in a significant overall decrease in these indicators (Table 3; S3C and S3D Fig). Also, high CB (but not CB%) was observed in GoR-BIOR, with a significant decrease over time for CB and a nearly significant increase in CB% (S3C and S3D Fig). For other datasets, the low values in the late 1960s (EGB-BIOR) or early 1980s (BoSFI) resulted in significant increase in CB towards the end of the observation period. Similarly, CB% increased significantly in GoFFI and Landsort, due to low values in 1980s-early 1990s (S3C and S3D Fig).

#### MMB and MMB%

For MMB, a shift from high values in 1980s-early 1990s to low and often out-of-control CuSum values in the late 1990s-2000s was observed in GoFFI, BIOR and Landsort datasets, while the opposite pattern occurred in Bornholm (Table 3; S3E Fig). For MMB%, the pattern was similar as for MMB, except GoR-BIOR dataset, where no out-of-control periods were detected (S3F Fig). Moreover, in both MMB and MMB%, an opposite shift from the low values in the 1980s to a consistently high out-of-control CuSum values during the last 15 years was observed for Askö and Bornholm (Table 3; S3F Fig).

#### Community ratios

The indicators based on community ratios (RotCla/Cop and Cla/Cop) changed in concert, due to the nature of their calculations (Table 3; S3G and S3H Fig). In the Gulf of Bothnia, no clear trends were observed, whereas a slight yet significant overall increase in RotCla/Cop occurred in the Åland Sea and northern Baltic proper (Askö). In the latter, a biphasic trend was observed, with a shift from low to high values in the mid-1990s and often out-of-control upper CuSum values in the late 1990s-2000s. By contrast, significant declining trends were observed in the Gulf of Finland and, for RotCla/Cop, in the Gulf of Riga. In the Landsort and Bornholm datasets, out-of-control upper CuSum values occurred in the mid-1990s and early 2000s, respectively (Table 3; S3G and S3H Fig).

#### MeanSize

Since the 1990s, the values declined in the northern Baltic proper and the Gulf of Finland as well as in the Bornholm, and in 1997–2007, the lower CuSum limits were violated in the ÅlandFI, GoFFI, Askö, Landsort and Bornholm datasets (Table 3; S3I Fig). The observed decrease of the mean zooplankter size (wet weight) in the community varied from 18% in the Bornholm to 57% in the Åland datasets estimated as a difference between the beginning and the end of the time series and using 5-year average values.

### Control charts with baselines estimated for RefCon_Fish_

#### TZA and TZB

In the Gulf of Bothnia, TZA exceeded CuSum UCL, whereas no appreciable changes in TZB were observed (Table 3; S3A and S3B Fig). By contrast, TZA and TZB exceeded CuSum LCL in GoR-BIOR and Bornholm datasets in the late 1990s and mid-2000s, respectively. That was also the case for TZB at Askö, which crossed the lower CuSum in the early 2000s.

#### CB and CB%

The greatest deviations in CB and CB% from RefCon_Fish_ values were recorded in Askö, GoR-BIOR and Bornholm datasets (Table 3; S3C and S3D Fig). For Askö and Bornholm, both CB and CB% started to decline synchronously in the mid-1990s and went below CuSum limits in 2000s (S3D Fig). In the Gulf of Riga, opposite trends were observed for CB and CB%, with CB declining in concert with that at Askö and Bornholm and CB% increasing and crossing upper CuSum in the mid-1990s. Moreover, in this dataset, CB% variability increased significantly after the mid-1990s (F-test; F_18,12_ = 3.3, p < 0.04) resulting in frequent violations of the Shewhart limits. Similar, albeit less pronounced and delayed increase in CB% leading to exceeded upper CuSum limit, were observed in GoFFI (end-1990s) and Landsort (mid-2000s). No appreciable changes occurred in the rest of the datasets.

#### MMB, MMB% and community ratios

CuSum trends for MMB and MMB% were largely opposite to those observed for CB and CB%, respectively (Table 3; S3E and S3F Fig). In particular, out-of-control MMB and MMB% values occurred in GoFFI (from early to the mid-2000s), GoR-BIOR (early 1990s onwards) and Bornholm (mid-1990s –mid-2000s) datasets. In Bornholm, it followed by a relatively short period of out-of-control CuSum UCL (S3E Fig). The decline was also observed for both ratios in GoFFI and GoR-BIOR, crossing the CuSum LCL in the early 2000s and mid-1990s, respectively. Around the late 1990s, MMB%, RotCla/Cop and Cla/Cop went over CuSum UCL in Askö, Bornholm and GoFFI datasets (S3F Fig). The changes in GoR-BIOR, Askö and Bornholm have coincided with the increased between-year variability and frequent violations of the Shewhart limits for all these indicators (S3E–S3H Fig). The underlying community changes that were behind these trends differed between the datasets. The downward trends in MMB-based indicators and community ratios in GoR-BIOR and GoFFI were related to the significantly decreasing rotifer and cladoceran biomass (data not shown), whereas the increases in MMB%, RotCla/Cop and Cla/Cop in Askö and Bornholm resulted primarily from the decreased copepod biomass (S3C Fig).

#### MeanSize

In ÅlandFI, GoFFI, Askö, Landsort and Bornholm datasets, lower CuSums for MeanSize exceeded their respective limits in the mid-1990s to early 2000s and had not returned to the baseline values until the end of the datasets (Table 3; S3I Fig). In EGB-BIOR, the MeanSize values were particularly low at the beginning of the data series (the early 1960s) increasing during the rest of the data period.

### Control charts with baselines estimated for RefCon_Chl_

#### TZA and TZB

For TZA, similar trends with increasing upper CuSum outside the RefCon_Chl_ period were observed for BoSFI, ÅlandFI, GoR-BIOR, Landsort, and, to some extent, Bornholm (Table 3; S3A Fig). For TZB, the out-of-control increase was recorded only for BoSFI (from the mid-1990s onwards) and, in early 1980s, in Bornholm (S3B Fig), whereas the decrease occurred in Askö (the mid-1990s), GoFFI and Bornholm (both in the mid-2000s). In EGB-BIOR, out-of-control low CuSums for both TZA and TZB were recorded in the 1960s. Also, high between-year variability following the RefCon_Chl_ period was observed for Bornholm TZA and TZB, with frequent violations of both upper and low Shewhart limits. Moreover, the variance for the post-reference period was significantly higher (F test; TZA: F_15,9_ = 5.28, p < 0.01; TZB: F_15,9_ = 5.39, p < 0.01).

#### CB and CB%

The greatest declines in CB and CB% with out-of-control CuSum values were recorded in Askö and Bornholm datasets from late the 1990s to early 2000s (Table 3; S3C and S3D Fig). Also, in ÅlandFI, the continuous decline in CB% starting shortly after the RefCon_Chl_ period, resulted violating both CuSum and Shewhart limits. The increase in CB exceeding CuSum UCL in the early 2000s was observed in BoSFI (S3C Fig). No appreciable changes were found for other datasets.

#### MMB, MMB% and community ratios

The most pronounced post-RefCon_Chl_ increase occurred in ÅlandFI (MMB, RotCla/Cop and Cla/Cop) and ÅlandFI, Askö and Bornholm (MMB%, RotCla/Cop and Cla/Cop) datasets (Table 3; S3E–S3H Fig). Also, high MMB and MMB% CuSum values were observed in GoR-BIOR prior RefCon_Chl_ (S3E Fig). The CuSum LCL for MMB and the community ratios were violated in GoFFI and EGB-BIOR datasets in the mid-2000s (S3E, S3G and S3H Fig), whereas MMB% and the community ratios declined in GoFFI, BoSFI and EGB-BIOR (S3F–S3H Fig). While all significantly violations of the CuSum and Shewhart UCLs were associated with significantly increasing variance (F test; p < 0.05 in all cases), the decreased MMB, MMB% and community ratios were never accompanied by the increased variance (F-test; p > 0.05 in all cases).

#### MeanSize

The MeanSize violated the Shewhart LCL at least once in 5 out of 9 datasets, and the CuSum LCLs were violated in ÅlandFI, GoFFI, Landsort, Askö and Bornholm datasets (Table 3; S3I Fig). The first out-of-control year for CuSum values ranged from 1995 (GoFFI) to 2001 (ÅlandFI), with no return to the baseline variability during the observation period.

### Abrupt shifts in community structure revealed by chronological clustering

The earliest breakpoint was detected for EGB-BIOR in the late 1960s. The change was related to the upward shift in total zooplankton stocks, including both copepods and cladocerans, with prevalence of cladocerans (Table 3; S3A, S3B, S3C, S3D and S3H Fig). In the early 1980s, a breakpoint was identified in the Åland Sea, albeit only at α levels of 0.05 and 0.1 (Table 4). Similar to the EGB-BIOR dataset, the increased total abundance due to the increased stocks of cladocerans and rotifers and, consequently, declining percentage of copepod biomass was responsible for this shift in the ÅlandFI (S3A, S3D, S3G and S3H Fig). More profound and significant changes in community structure were detected during the mid-1990s in the northern Baltic proper and the Gulf of Finland, but also in the eastern Gotland basin and Bornholm when using α levels of 0.05 and 0.1 (Table 4). In the late 1990s, the breakpoint in the Bornholm was identified at all α levels. These changes were related to declining zooplankton stocks with concomitant changes in the community structure. The structural changes were, however, different between the offshore northern Baltic together with the Gulf of Finland, where cladoceran biomass declined, and coastal northern Baltic proper together with Bornholm basin, where the decline was mostly attributed to the copepod biomass (Table 3; S3C, S3D and S3H Fig). Finally, in the early 2000s, the less pronounced shifts in the Bothnian and Åland seas and in the Bornholm were identified with lower α levels (Table 4).

**Table 4:** Shifts in zooplankton community structure (year) that were detected for different α levels using the indicator time series (only entire datasets with more than 12 years of observations were considered). No community shifts were detected for the Bothnian Bay (BoBFI) data. Years that were consistently detected at all levels of α are in bold.

Data sets	α = 0.01	α = 0.05	α = 0.1
BoSFI			2002
ÅlandFI		1983	1983, 2003
GoFFI	**1995**	**1995**	**1995**
Landsort	**1995**	**1995**	**1995**
Askö	**1996**	**1996**	**1996**
GoR-BIOR		1999	
EGB-BIOR	**1967**	**1967**	**1967**, 1994, 1997
Bornholm	**1999**	1994, **1999**, 2003	1994, **1999**, 2003

### Covariation among the indicators

There were significant correlations among the indicators, with substantial differences among the datasets (S2 Table, S4 Fig). Due to the nature of the indicator calculations, the strongest and ubiquitous correlations were observed for RotCla/Cop, Cla/Cop and CB%, which are the indicators reflecting proportions of copepods and cladocerans, the two major groups contributing to the community biomass. As cladocerans contribute heavily to MMB and MMB%, these indicators were also strongly correlating with the indicators reflecting proportions of cladocerans and copepods. TZA and TZB exhibited moderate to strong intercorrelations in all datasets except BoBFI, whereas Pearson *r* for MeanSize was most variable, with significant moderate to strong positive correlations observed for CB and/or CB% in BoBFI, BoSFI, Askö, and Bornholm datasets.

### Linking indicators to the reference conditions

Indicators that were significantly associated with in-control zooplankton community state for RefCon_Chl_ and RefCon_Fish_ were identified by logistic regressions (Table 5). MeanSize was a significant predictor in >50% and 75% of the best-fit models for RefCon_Chl_ and RefCon_Fish_, respectively. The best-fit models that did not include MeanSize frequently identified a combination of TZA and TZB as negative and positive predictors, respectively (Table 5, models 7, 12–13), implying a positive effect of MeanSize on the response variable. The latter was always observed in the alternative models, albeit with lower fit. The main difference between the sets of the models for RefCon_Chl_ and RefCon_Fish_ was that the latter consistently included variables describing total zooplankton stock size (TZA, TZB or both) as positive predictors, unlike the former that included variables related to community structure (percentages of main groups and/or ratios). Also, in RefCon_Fish_ models, in contrast to the RefCon_Chl_ models, both TZA and TZB effects were predominantly positive.

**Table 5:** Winning logistic models for prediction of zooplankton community structure being in the reference state for RefCon_Chl_ and RefCon_Fish_. When equally strong models were found for the same dataset, their AIC values are provided. Only significant models are shown; significant effects are in bold. Correct classification percentage and odds ratio are used for model accuracy evaluation.

Datasets	AIC	Predictors	β	SE	Wald statistic	*p*	Log odds ratio for the model	Correct classification, %		
								In-control	Out-of-control	Overall
**RefCon**_**Chl**_										
1. BoSFI		TZA	-1.42	0.58	5.99	**0.014**	2.1	80	67	74
2. Åland		CB%	2.42	1.07	5.05	**0.024**	3.6	67	95	88
3. GoFFI		MMB%	2.61	1.15	5.10	**0.023**	3.7	88	85	86
		MeanSize	2.63	1.14	5.81	**0.012**				
4. Askö		MMB%	-3.03	1.01	8.94	**0.002**	3.6	79	91	86
5. Landsort	*Model A*: 27.3	TZB	-1.25	0.74	2.85	0.092	2.6	75	81	79
		MeanSize	3.20	1.25	6.49	**0.010**				
	*Model B*: 27.4	TZA	-1.01	0.60	2.73	0.092	2.3	67	82	75
		MeanSzie	2.31	0.98	5.50	**0.018**				
6. GoR-BIOR		TZA	-1.46	0.49	7.02	**0.007**	1.9	81	60	75
7. EGB-BIOR	*Model A*: 66.9	TZA	-2.13	1.20	3.14	0.076	1.6	70	66	67
		TZB	2.75	1.20	5.21	**0.022**				
	*Model B*: 67.2	MMB	0.59	0.33	3.12	0.077	1.7	71	67	68
		MeanSize	0.71	0.21	4.70	**0.042**				
8. Bornholm		TZA	1.41	0.70	3.96	**0.046**	2.6	62	90	78
		MMB	-1.93	0.82	5.48	**0.019**				
9. EGB-BIOR, Bornholm	*Model A*: 109.2	TZA	0.51	0.24	4.25	**0.039**	1.2	54	73	65
		MeanSize	0.78	0.27	8.06	**0.004**				
	*Model B*: 109.7	TZB	0.47	0.23	3.86	**0.049**	1.5	59	76	69
		MeanSize	0.59	0.26	4.81	**0.028**				
10. Landsort, Askö, Bornholm		TZB	2.17	0.69	9.78	**0.002**	2.0	67	79	74
		RotCla/Cop	-1.98	0.59	10.99	**0.001**				
		MeanSize	1.09	0.37	8.58	**0.003**				
11. GoFFI, GoR-BIOR		TZA	-3.46	1.07	10.35	**0.001**	2.3	87	61	77
		MMB	4.76	1.83	6.77	**0.009**				
12. GoFFI, GoR-BIOR, EGB-BIOR		TZA	-2.32	0.66	12.21	**0.001**	1.5	80	54	69
		TZB	2.49	0.66	14.08	**0.001**				
13. ÅlandFI, Landsort, Askö		TZA	-0.97	0.46	4.40	**0.035**	2.5	66	87	79
		TZB	1.65	0.53	9.42	**0.002**				
		Cla/Cop	1.98	0.73	7.23	**0.007**				
**RefCon**_**Fish**_										
14. GoFFI		MeanSize	1.98	0.78	6.40	**0.011**	3.7	87	86	86
15. Askö		MeanSize	2.63	0.89	8.69	**0.003**	3.2	81	84	83
16. GoR-BIOR		TZA	1.18	0.50	5.71	**0.017**	1.2	43	82	69
17. EGB-BIOR		MeanSize	1.32	0.43	9.04	**0.002**	2.0	73	73	73
18. Bornholm		CB	1.97	0.72	7.45	**0.006**	2.9	81	81	81
19. Landsort, Askö		MMB	0.91	0.45	3.97	**0.046**	1.6	62	76	70
		RotCla/Cop	-1.50	0.49	9.20	**0.002**				
		MeanSize	0.50	0.3	6.27	**0.032**				
20. BoSFI, ÅlandFI, Landsort, Askö		TZA	-0.74	0.33	4.97	**0.025**	1.4	53	79	68
		MMB	1.36	0.50	7.28	**0.006**				
		RotCla/Cop	-1.20	0.36	11.03	**0.001**				
21. Landsort, Askö, EGB-BIOR, Bornholm		CB	0.43	0.20	4.39	**0.036**	1.9	65	79	72
		MeanSize	0.81	0.22	13.08	**0.001**				
22. GoR-BIOR, EGB-BIOR		TZA	0.54	0.24	4.73	**0.029**	1.7	67	73	70
		MeanSize	0.65	0.27	5.78	**0.016**				
23. GoFFI, GoR-BIOR, EGB-BIOR, Landsort		MMB	0.39	0.19	4.08	**0.043**	1.3	56	74	66
		MeanSize	0.45	0.19	9.09	**0.003**				
**RefCon**_**Chl**_										
24. ÅlandFI, Landsort, Askö GoFFI, GoR-BIOR, EGB-BIOR		TZB	0.32	0.16	4.12	**0.022**	1.5	54	80	69
		MeanSize	0.54	0.17	9.67	**0.001**				
25. ÅlandFI, Landsort, Askö GoFFI, GoR-BIOR, EGB-BIOR, Bornholm		MMB	0.69	0.25	7.46	**0.006**	1.3	59	72	67
		RotCla/Cop	-0.56	0.25	4.70	**0.030**				
		MeanSize	0.70	0.16	19.10	**<0.000**				

The overall prediction accuracy of the models was 65–88% and 66–86% for the RefCon_Chl_ and RefCon_Fish_ models, respectively (Table 5; S5 Fig). The sensitivity, i.e., the proportion of cases (years) correctly identified by the model as being within the reference conditions, was similar between RefCon_Chl_ and RefCon_Fish_ models. By contrast, specificity, i.e., the proportion of cases correctly identified as being outside the reference conditions, was significantly higher in the RefCon_Fish_ models, with lower between-model variability (72–86%; Wilcoxon matched-pairs signed rank test; p < 0.004; S5 Fig).

## Discussion

### High spatial variability of zooplankton and its indicators

Ecologists and managers have long recognized the challenges imposed by the inherent spatial complexity of aquatic communities for indicator development within MSFD. While studies on long-term zooplankton dynamics in some Baltic areas have received considerable attention [22, 28, 40, 59; 78], the temporal variability of zooplankton across the sea remains much less understood. The need for indicators that would be equally applicable in different areas highlights this concern. Our examination of various metrics reflecting zooplankton community dynamics revealed high variability among the basins of the Baltic Sea, typical for marine estuaries [35]. Total zooplankton stocks were highest in the Baltic proper and the adjacent Gulfs of Riga and Finland, mostly due to the higher contribution of cladocerans but also greater copepod stocks (Fig 3). The cross-Baltic variability of the mean zooplankter size reflects relative contribution of both large copepods (e.g., *Limnocalanus macrurus* in the Bay of Bothnia) and large size classess of cladocerans (e.g., *Evadne nordmanni* and *Bosmina maritima* in the Gulf of Riga and the eastern Gotland basin). These differences emphasize the need for non-taxonomic zooplankton indicators that would represent common features regarding the food web functioning in the pelagia, i.e., maintaining energy raceways from primary producers to higher trophic levels.

### Trends and shifts in zooplankton community structure

Both monotonous trends (S3 Fig) and sudden shifts (Table 4) were detected during the past decades in virtually all datasets; moreover, these changes for specific indicators varied in their direction and timing among the datasets. In all areas, except the Bothnian Bay, chronological clustering identified shifts in zooplankton community structure and stock size, with the earliest shift observed in the late 1960s, and the most profound pan-Baltic changes occurring in mid- to late 1990s (Table 4); the latter is in agreement with the best documented regime shift in the central Baltic [79]. However, the taxonomic and structural changes underlying these shifts differed among the areas; as a result, most indicators showed both increases and decreases over time depending on the dataset. The most consistent trends were observed for MeanSize that significantly decreased in the northern Baltic proper and the adjacent areas (Åland Sea and the Gulf of Finland) as well as in the Bornholm basin (S3 Fig) due to decreasing stocks of larger copepods, such as *Limnocalanus macrurus* in the north and *Pseudocalanus* spp. in Bornholm basin, respectively [78]. The observed absolute decrease of the mean zooplankter body weight varied from 18% in the Bornholm to 57% in the Åland datasets. Such profound and consistent throughout the ecosystem decline in the body size of pelagic grazers and fish prey have strong implications for both grazing capacity of zooplankton community and fish feeding conditions.

### Detecting out-of-control periods with control charts

The combination of Shewhart and CuSum control charts provides a useful tool in the analysis of both sudden deviations and persistent small changes in zooplankton metrics. In each data set, at least one indicator was found to cross control limits regardless whether the acceptable background variability of the indicator was based on the entire dataset or on the reference period only (Table 3). When the baseline variability was set based on the entire dataset, the indicators that violated their control limits most frequently were MeanSize, TZA, MMB%, MMB and the community ratios (Table 3). The datasets with the highest number of the indicators violating their control limits were GoFFI, Landsort, Bornholm and EGB-BIOR (Table 3). When reference periods based of the existing EQR for chlorophyll and fish condition were used in setting up the baseline variability, the number of the violations were fewer for most of the indicators compared to those identified when using the entire dataset variability. The most characteristic violations were observed for MeanSize. When outside any reference period, MeanSize crossed lower control limit in all datasets except BoSFI and GoR-BIOR; this was observed more frequently for the RefCon_Fish_- than for RefCon_Chl_-based evaluations (Table 3). Behavior of the indicators reflecting total zooplankton stock (TZA and TZB) differed among the areas, with values indicating suboptimal fish feeding conditions in the coastal areas of the western and eastern Baltic proper and the Bornholm basin. The differences were also apparent with regard to the eutrophication degree, with TZA, TZB and MeanSize values indicating increased total abundance or decreased biomass in combination with decreased body size in zooplankton in most of the datasets (Table 3). The behavior of indicators reflecting contribution of copepods, small-sized zooplankters and community ratios was also informative, albeit only for some datasets, depending on the area-specific community structure and natural prevalence of cladocerans or copepods (S3 Fig). Most of the violations occurred in 1990s, and, in many cases, the deviations from in-control state have escalated dramatically toward the end of the time series and never returned to the baseline levels, particularly in the RefCon_Fish_-based evaluations (e.g., MeanSize, Cla/Cop, CB%, MMB%; Table 3). Notably, in the longest dataset (EGB-BIOR, ~50 years), the most pronounced period of sub-GES zooplankton state appeared to occur in the 1960s, when both TZA and TZB were exceptionally low, reflecting low stocks of copepods and cladocerans (S3 Fig). While we can only speculate about the driving forces behind the increase in zooplankton stocks during the late 1960s to 1970s, it is clear that this increase and the following period of high zooplankton stocks coincided with good feeding conditions for herring and sprat [38,79,80]. Finally, as with any other biological data, the uncertainty related to sampling and data analysis comparability over decades remains important for compiling long-term data sets. With regard to the EGB-BIOR data, one should keeep in mind that the sampling and analysis methods behind this dataset deviated most from the HELCOM guidelines that were followed more closely by other laboratiories (Table 1, S1 Table), which complicates the interpretation of the indicator trends.

### Combining indicators to predict GES

We demonstrated the diagnostic yield of the putative indicators using logistic regressions that identified indicators for predicting whether zooplankton community is within variability typical of RefCon_Chl_ or RefCon_Fish_ conditions. Each of the 25 regression models achieved a high level of statistical proficiency with three or fewer predictive variables. The model reliability was moderate to high, 65–88% and 66–86% for the RefCon_Chl_ and RefCon_Fish_ models, respectively (Table 5). The model sensitivity was similar between RefCon_Chl_ and RefCon_Fish_ models, whereas model specificity was significantly higher in the RefCon_Fish_ models, with lower between-model variability (~80%; S5 Fig). Thus, the RefCon_Fish_ models can predict equally well cases both within and outside the fish feeding reference conditions whereas the RefCon_Chl_ models are reliable for predicting in-GES cases. In all models, MeanSize was the most common significant predictor, contributing to >50% and 75% of the models for RefCon_Chl_ and RefCon_Fish_, respectively (Table 5). In all cases, the probability of falling outside the reference state increased with decreasing body size of a zooplankter in the community. Alternatively, a combination of TZA and TZB as negative and positive predictors, respectively, was observed, implicating a positive effect of MeanSize on the probability of zooplankton community being in the reference state. In some basin-specific models, also other metrics, such as copepod biomass (CB), the contribution of copepods to the total zooplankton biomass (CB%), the biomass-based community ratios or contribution of microphagous groups (MMB%), were equally good or even superior. For example, CB was a single positive predictor for fish feeding conditions in the Bornholm basin, whereas MMB% was a single negative predictor for the coastal northern Baltic proper (Askö); both models had high classification accuracy (Table 5).

### Overlapping RefCon_Chl_ and RefCon_Fish_ conditions

Delineating zooplankton indicators of the in-GES state between the RefCon_Chl_ and RefCon_Fish_ is particularly challenging because these periods largely overlap in our datasets (Fig 2). As a result, the zooplankton datasets used to train the models, overlap as well. Moreover, one has to keep in mind that the reference periods that represent pelagic food web “not measurably affected by eutrophication” were defined using existing EQR for chlorophyll. In the Baltic Sea, the documented chlorophyll data extend back to the 1980s, rarely 1970s, which means that they are not likely to cover truly non-eutrophic conditions [61,81]. Thus, for all datasets (except, perhaps, the Gulf of Bothnia), the baselines corresponding to RefCon_Chl_ are, in fact, likely to represent mesotrophic to eutrophic communities that were typical for the Baltic Sea in the 1970s-80s [82]. Therefore, in addition to the constraints related to the data availability, the overlap between these conditions may have occurred in the Baltic Sea, similar to other systems, where moderately eutrophied conditions were beneficial for fish production [83,84]. The latter can explain why the indicators for RefCon_Chl_ and RefCon_Fish_ are similar. However, in the RefCon_Fish_ models, the indicators describing total zooplankton stock size (TZA, TZB or both) contributed as positive predictors, unlike the RefCon_Chl_ models where indicators related to community structure (i.e., percentages of main groups and/or ratios) were more significant (Table 5). These differences in the relative importance of the predictors are indicative of the structural and functional properties of a food web with high energy transfer efficiency *vs*. food web not measurably affected by eutrophication [85].

### Conclusions and Future Directions

**Fig 4:**
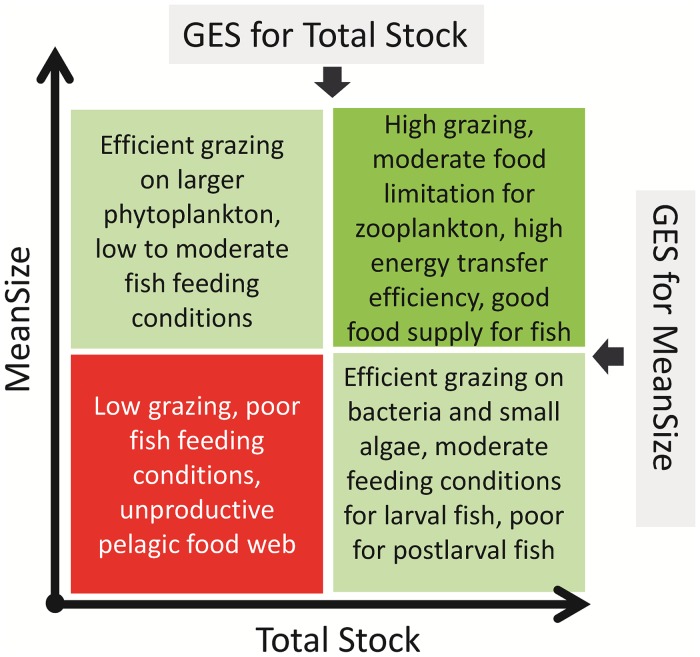
Conceptual diagram for MSTS, a two-dimensional indicator comprised by total stock (TZA or TZB) and MeanSize. The green area represents in-GES condition, orange areas represent sub-GES conditions where only one of the two parameters is adequate, and the red area represents sub-GES conditions where both parameters fail.

On the basis of our operational definition of GES, we propose mean body mass of zooplankter in the community (MeanSize) in combination with zooplankton stock measured as either abundance (TZA) or biomass (TZB) to be applicable as an integrated indicator within the Descriptor 4 in the Baltic Sea. These metrics performed best in predicting zooplankton being in-GES when considering all datasets evaluated and can be integrated as a single two-dimensional indicator representing the mean size and total stock (MSTS) of zooplankton (Fig 4). The rationale for MSTS is as follows. High standing stocks of zooplankton composed by larger organisms have a higher capacity for transfer of primary production to fish production (i.e., higher energy transfer efficiency). By contrast, the dominance of small-sized organisms indicates the prevalence of microbial prey and thus inefficient energy transfer due to losses in microbial loop. Thus, abundant zooplankton with high mean individual size would represent both favorable fish feeding conditions and high grazing potential. All other combinations of zooplankton stock and individual size would be suboptimal and imply food web limitations regarding energy transfer from primary producers to higher trophic levels and poorer food availability for planktivorous fish. Of course, these conclusions are based on the zooplankton data representing only the growth period (June–September); a further evaluation is required to understand indicator properties of zooplankton during other seasons. Nevertheless, our evaluation suggests that in several basins of the Baltic Sea, such as Åland Sea, northern and southern Baltic proper and major gulfs (Gulf of Finland and Gulf of Riga), zooplankton communities currently appear to be out-of-GES, being comprised by smaller zooplankters and having lower total abundance or biomass compared to the communities during the reference conditions. However, the changes in the taxonomic structure underlying these trends vary widely across the sea basins.

In future work, MSTS should be validated for all Baltic Sea areas where zooplankton monitoring is conducted. In particular, its applicability in the Western Baltic Sea, much of the Eastern, South-Eastern, and Southern Baltic, as well as southern and eastern parts of the Gulf of Finland, needs to be tested before MSTS can be applied in these assessment units. In addition to MSTS, region-specific supporting indicators can be considered based on our findings showing the adequate performance of other community metrics in the binary models. Also, temperature- and salinity-induced changes in MSTS need to be evaluated and, if relevant and significant, they need to be accounted for in the indicator-based assessment of eutrophication effects on pelagic food webs. Moreover, the outcome of MSTS-based assessment needs to be cross-checked with other food web indicators within Descriptor 4 as well as eutrophication status according to current ecological status assessment in the specific assessment units.

Methodologically solid monitoring programme is a prerequisite to interpret, evaluate and predict the state of the zooplankton communities. To this end, harmonization of sampling and analytical methods for zooplankton analysis in the Baltic Sea is essential for the application of the unified indicator approach across the sea basins. For MSTS validation, particularly valuable are long data series with high taxonomic resolution that allows accurate individual size and total biomass assessment. For data-poor areas, a cross-basin surveillance program would help establishing indicator baselines by identifying neighbouring data-sufficient areas with similar communities. However, the ultimate goal of the monitoring is to generate sufficiently long data sets using consistent methods and gears, if we are to detect meaningful changes in zooplankton communities and to provide scientific advice on ecosystem management.

## Supporting Information

S1 Fig Seasonal development of (a) phytoplankton biomass, (b) zooplankton biomass, and (c) estimated food consumption by zooplanktivorous fish in the Baltic Sea, northern Baltic proper. (PDF)

S2 Fig Variation in coefficient of variation (CV%, mean±SD) for different indicators before and after Box-Cox transformation. The transformation significantly decreased variance for all indicators except CB% and MMB% (Wilcoxon signed rank test, p < 0.015). The indicator-specific CV% values correspond to (A) the entire time series, (B) the reference period based on Chl *a* values, and (C) the reference period based on the WAA of planktivorous fish. See Table 2 for indicator abbreviations and Fig 2 for the time definition of the reference periods. Note the differences in Y-scales between the panels.
(PDF)

S3 Fig Control charts for all indicators with baselines estimated for the entire data sets (upper panel), RefConChl (middle panel), and RefConFish (bottom panel). Upper (red line) and lower (blue line) DI-CuSums and Shewhart z-scores (open circles) are shown on the left and right y-axes, respectively. A shaded area represents in-control Shewhart limits and dashed lines represent upper (UCL) and lower (LCL) CuSum limits. The upper and lower control limits, were defined as either 99%-CIs around the mean values (for baseline based on an entire dataset) or using a conservative approach of ±3σ and ±5σ for Shewhart and CuSum control limits, respectively (for baseline based on either RefConFish or RefConChl). The p-values indicate significance for the non-parametric Mann—Kendall (Kendall, 1975) test for a monotonic downward or upward trend. (A) TZA, (B) TZB, (C) CB, (D) CB%, (E) MMB, (F) MMB%, (G) RotCla/Cop, (H) Cla/Cop, and (I) MeanSize. See Table 1 for details on the data origin, Table 2 for the indicator description, and Table 3 for the synthesis of the violations presented in S3 Fig.
(PDF)

S4 Fig Pairplot for the correlations between the indicators for all datasets combined. (PDF)

S5 Fig Classification accuracy for binary logistic models predicting zooplankton community structure being in the reference state (*in-control*) or not (*out-of-control*) for RefCon_Chl_ (A) and RefCon_Fish_ (B); see Table 4 for the list of models and their specifications. Significantly higher and less variable prediction accuracy was obtained for identification of zooplankton community structure as being outside of the reference values in the RefCon_Fish_ models (B; Wilcoxon matched-pairs signed rank test, p < 0.004).
(PDF)

S1 Table Details for zooplankton sampling and analysis methods employed in the national laboratories. (PDF)

S2 Table Pearson *r* correlations among the indicators in each dataset. Significant correlations at p < 0.05 are in bold; *n*–number of samples (i.e., the number of years included in the dataset). See Tables 1 and 2 for indicator and dataset descriptions.
(PDF)
