# Supplementary material for: Indicator Properties of Baltic Zooplankton for Classification of Environmental Status within Marine Strategy Framework Directive
Source: PLoS One. 2016 Jul 13;11(7):e0158326. doi: 10.1371/journal.pone.0158326 (PMC4943737; doi:10.1371/journal.pone.0158326)
Supplement: S1 Fig — (PDF) [file pone.0158326.s001.pdf]

PLoS One │ Supporting Information

Indicator properties of Baltic zooplankton for classification of environmental status within Marine Strategy Framework Directive

Elena Gorokhova[1*], Maiju Lehtiniemi[2], Lutz Postel[3], Gunta Rubene[4], Callis Amid[1], Jurate Lesutiene[5], Laura Uusitalo[2], Solvita Strake[6] and Natalja Demereckiene[7]

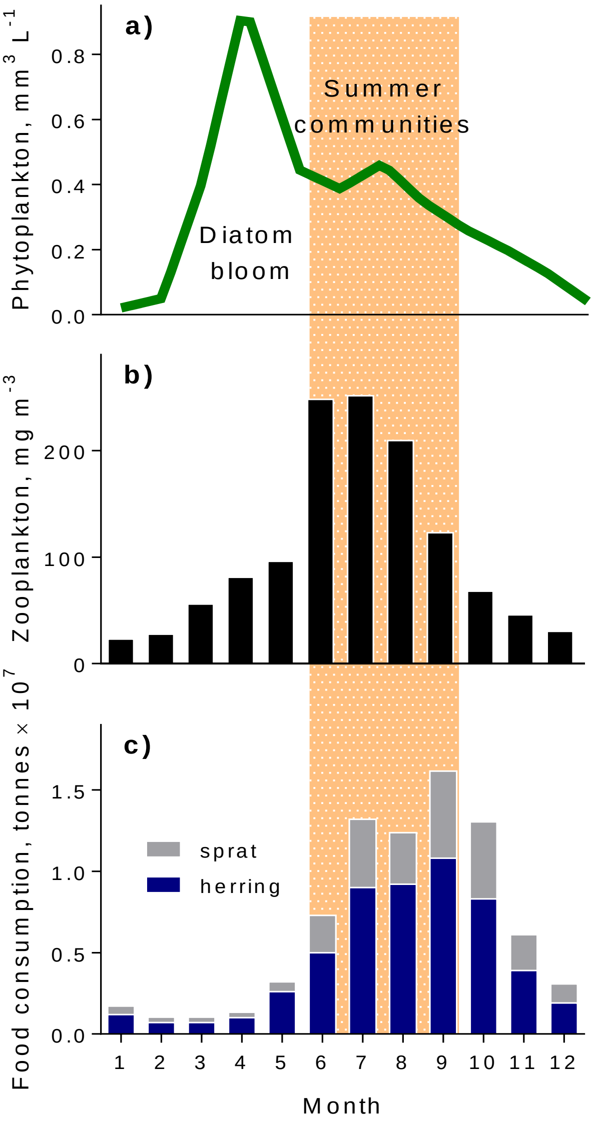

S1 Fig. Seasonal development of (a) phytoplankton biomass, (b) zooplankton biomass, and (c) estimated food consumption by zooplanktivorous fish in the Baltic Sea, northern

Baltic proper. The seasonal dynamics is shown to indicate the relative importance of the data period (June to September; highlighted in yellow) used in this study.

In spring, the diatom bloom remains largely ungrazed by zooplankton, because of the low stocks of the latter, and the diatom production settles relatively quickly to the bottom. In June–September, zooplankton stocks are at maximum exerting grazing control on summer phytoplankton communities. During the same time, the food consumption by fish is also at its maximum because of the high abundance of fish larvae after spawning in May-June. Moreover, a part of herring population spawns in August, which implies that zooplankton abundance and community structure during August-September are also important for fish recruitment. The phytoplankton and zooplankton data are long-term mean values (1992–2011) for the Askö area (station B1); the phytoplankton data were retrieved from the SHARK database (Swedish Meteorological and Hydrological Institute; www.smhi.se). The estimated food consumption by zooplanktivorous fish are from Arrhenius and Hansson (1993). The figure is modified from Karlson et al. (2015).

References

Arrhenius, F., and S. Hansson. 1993. Food consumption of larval, young and adult herring and sprat in the Baltic Sea. Marine Ecology Progress Series 96: 125–137.

Karlson AML, Duberg J, Motwani NH, Hogfors H, Klawonn I, Ploug H, Sveden JB, Garbaras A, Sundelin B, Hajdu S, Larsson U, Elmgren R, Gorokhova E (2015) Nitrogen fixation by cyanobacteria stimulates production in Baltic food-webs. AMBIO 44 (Suppl. 3): S413–S426
