# Supplementary material for: Indicator Properties of Baltic Zooplankton for Classification of Environmental Status within Marine Strategy Framework Directive
Source: PLoS One. 2016 Jul 13;11(7):e0158326. doi: 10.1371/journal.pone.0158326 (PMC4943737; doi:10.1371/journal.pone.0158326)
Supplement: S2 Fig — The transformation significantly decreased variance for all indicators except CB% and MMB% (Wilcoxon signed rank test, p < 0.015). The indicator-specific CV% values correspond to (A) the entire time series, (B) the reference period based on Chl a values, and (C) the reference period based on the WAA of planktivorous fish. See Table 2 for indicator abbreviations and Fig 2 for the time definition of the reference periods. Note the differences in Y-scales between the panels. (PDF) [file pone.0158326.s002.pdf]

PLoS One │ Supporting Information

Indicator properties of Baltic zooplankton for classification of environmental status within Marine Strategy Framework Directive

Elena Gorokhova[1*], Maiju Lehtiniemi[2], Lutz Postel[3], Gunta Rubene[4], Callis Amid[1], Jurate Lesutiene[5], Laura Uusitalo[2], Solvita Strake[6] and Natalja Demereckiene[7]

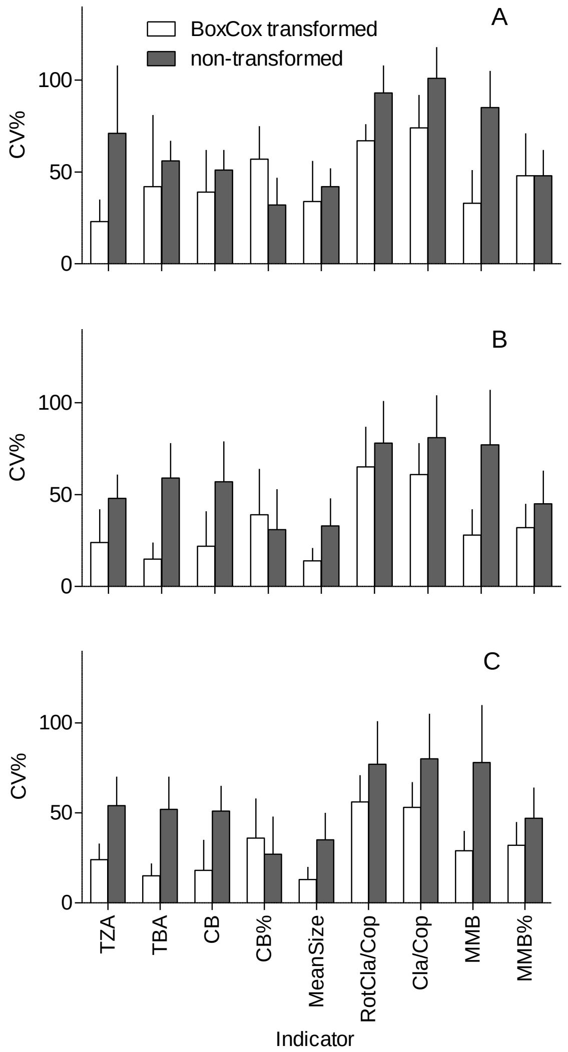

S2 Fig. Variation in coefficient of variation (CV%, mean±SD) for different indicators calculated using CV% for each specific indicator and dataset before and after Box-Cox transformation. The transformation significantly decreased variance for all indicators except CB% and MMB% (Wilcoxon signed rank test, $p < 0.015$). The indicator-specific CV% values correspond to (A) the entire time series, (B) the reference condition period based on Chl a values, and (C) the reference condition period based on the WAA of planktivorous fish. See Table 2 for indicator abbreviations and Figure 2 for the time definition of the reference periods.
