# Supplementary material for: Indicator Properties of Baltic Zooplankton for Classification of Environmental Status within Marine Strategy Framework Directive
Source: PLoS One. 2016 Jul 13;11(7):e0158326. doi: 10.1371/journal.pone.0158326 (PMC4943737; doi:10.1371/journal.pone.0158326)

PLoS One │ Supporting Information

Indicator properties of Baltic zooplankton for classification of environmental status within Marine Strategy Framework Directive

Elena Gorokhova[1*], Maiju Lehtiniemi[2], Lutz Postel[3], Gunta Rubene[4], Callis Amid[1], Jurate Lesutiene[5], Laura Uusitalo[2], Solvita Strake[6] and Natalja Demereckiene[7]

S3 Fig. Control charts for all indicators with baselines estimated for the entire data sets (upper panel), $\text{RefCon}_{\text{Chl}}$ (middle panel), and $\text{RefCon}_{\text{Fish}}$ (bottom panel). Upper (red line) and lower (blue line) DI-CuSums and Shewhart *z*-scores (open circles) are shown on the left and right y-axes, respectively. Grey area represents in-control Shewhart limits and dashed lines represent upper (UCL) and lower (LCL) CuSum limits. The upper and lower control limits, were defined as either 99%-CIs around the mean values (for baseline based on an entire dataset), or using a conservative approach of $\pm 3\sigma$ and $\pm 5\sigma$ for Shewhart and CuSum control limits, respectively (for baseline based on either $\text{RefCon}_{\text{Fish}}$ or $\text{RefCon}_{\text{Chl}}$). The p-values indicate significance for the non-parametric Mann–Kendall (Kendall, 1975) test for a monotonic downward or upward trend. (A) TZA, (B) TZB, (C) CB, (D) CB%, (E) MMB, (F) MMB%, (G) RotCla/Cop, (H) Cla/Cop, and (I) MeanSize. See Table 1 for details on the data origin and Table 2 for the indicator description.

Figure S3, A

TZA, Entire dataset period

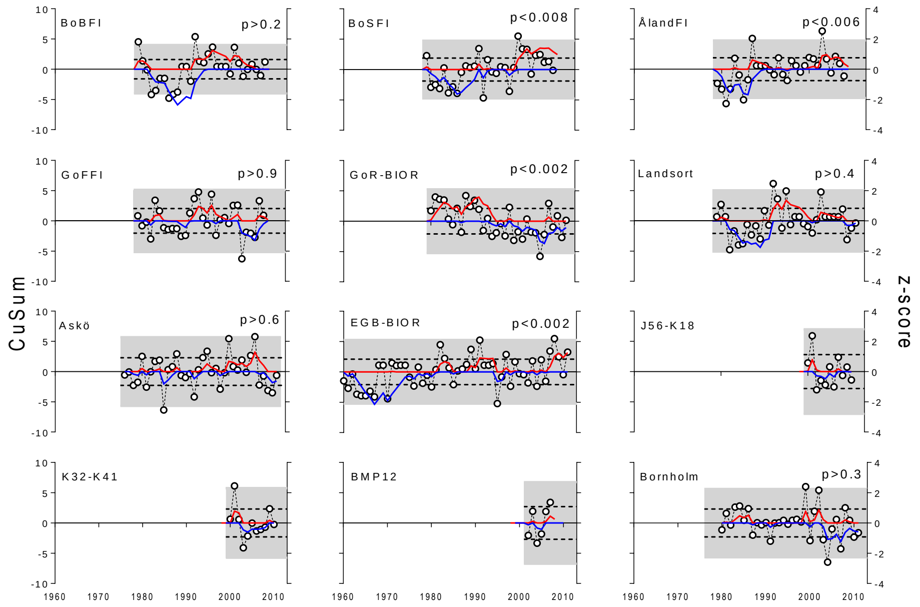

TZA, $RefCon_{Fish}$

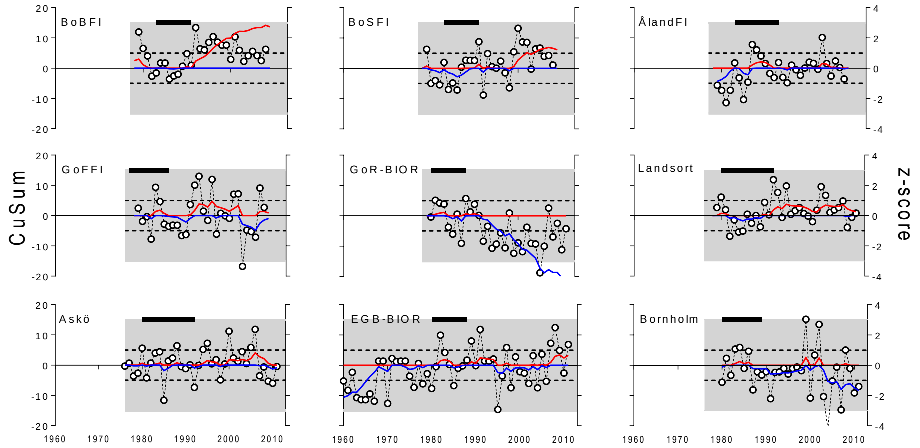

TZA, $RefCon_{Chl}$

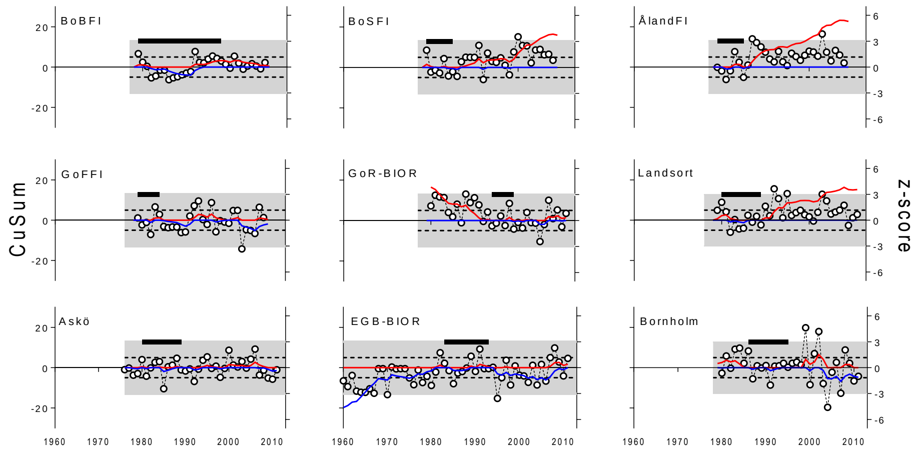

Figure S3, B

TZB, Entire dataset period

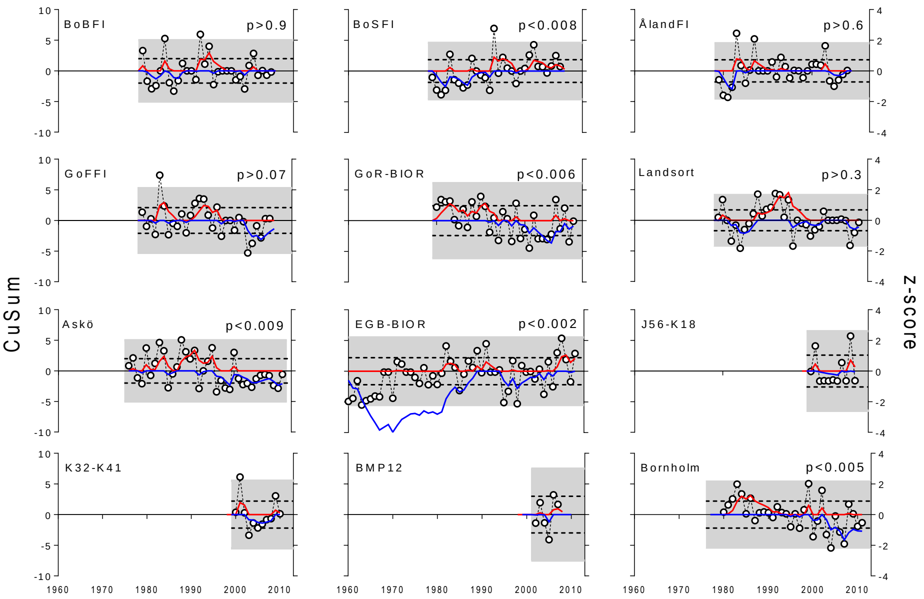

TZB, $RefCon_{Fish}$

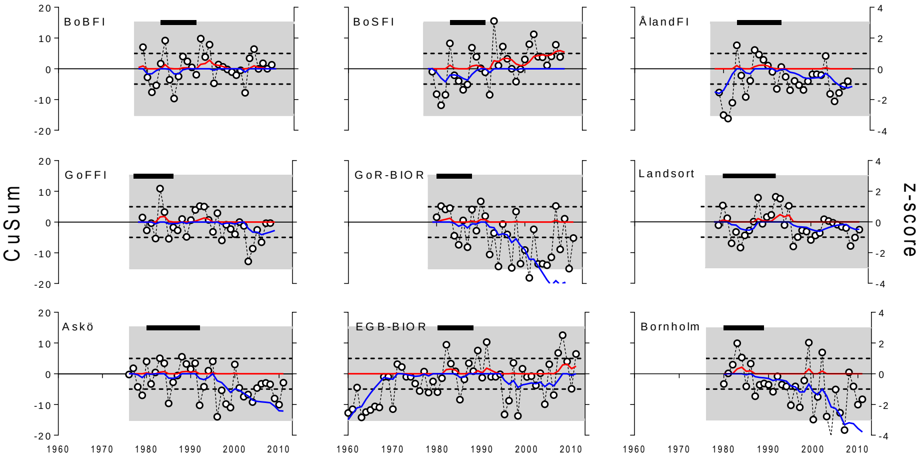

TZB, $RefCon_{Chl}$

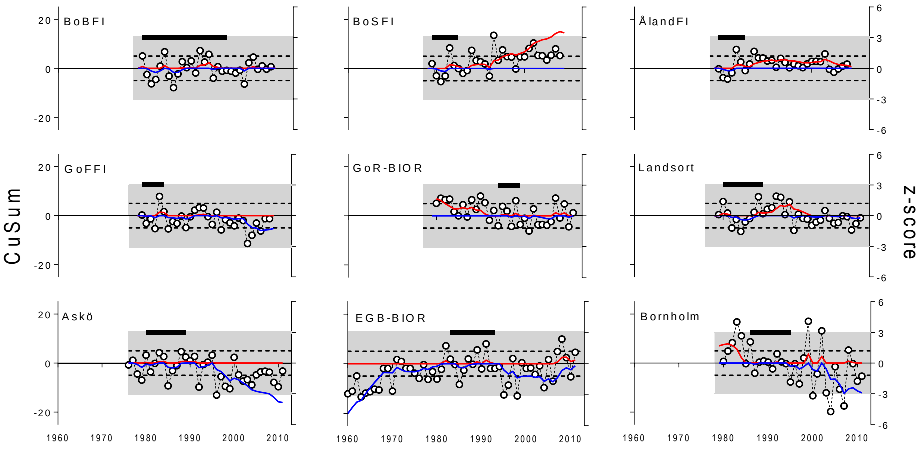

Figure S3, C

CB, Entire dataset period

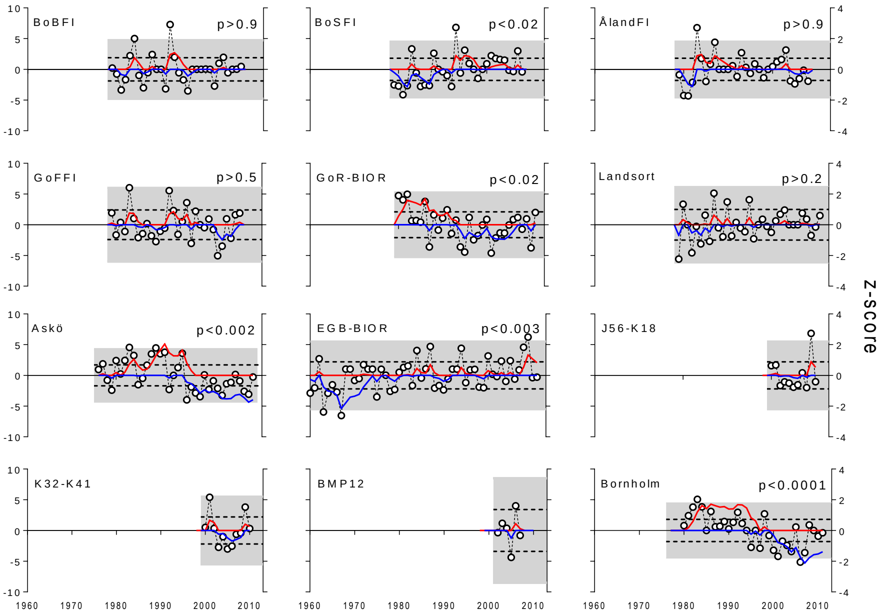

CB, $RefCon_{Fish}$

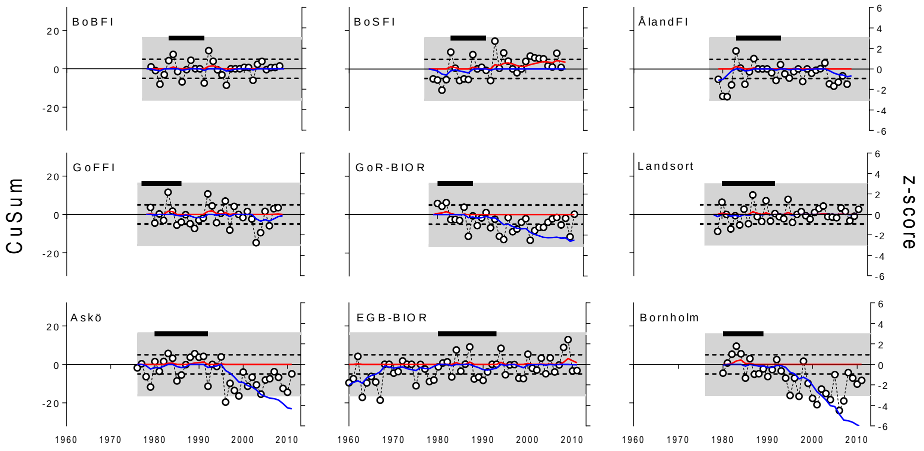

CB, $RefCon_{Chl}$

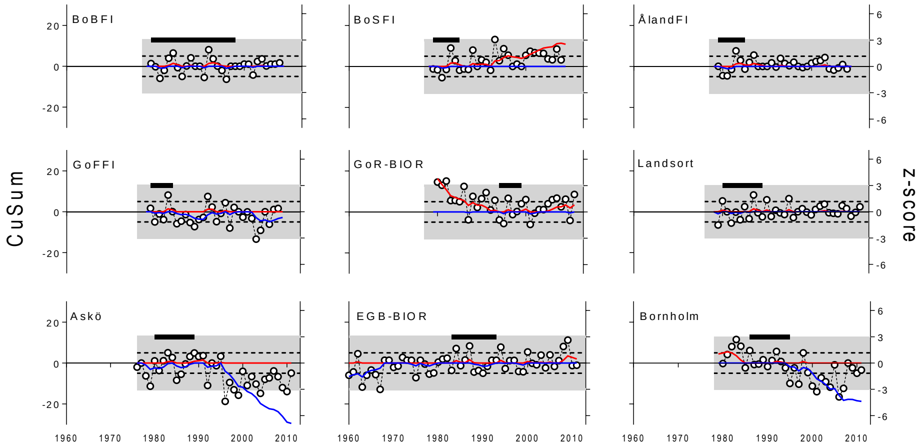

Figure S3, D

% CB, Entire dataset period

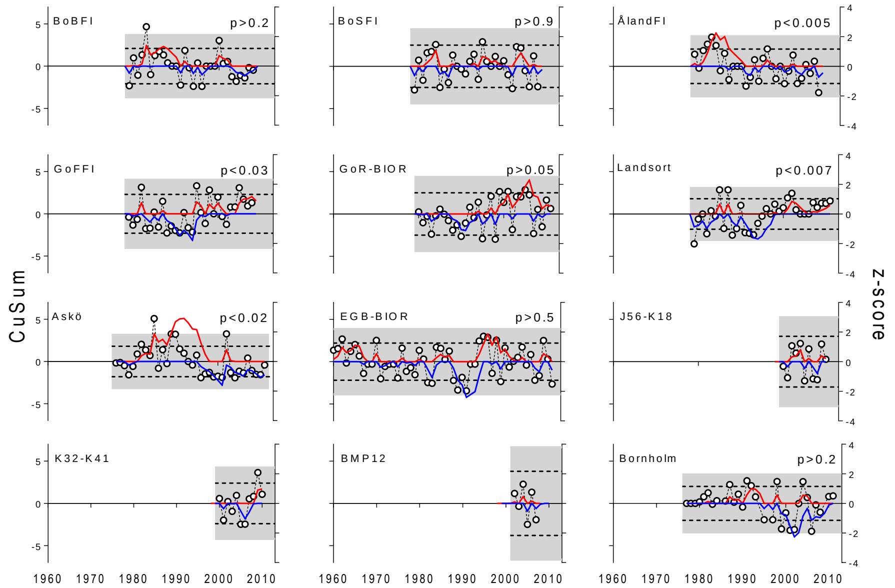

% CB, RefCon$_{Fish}$

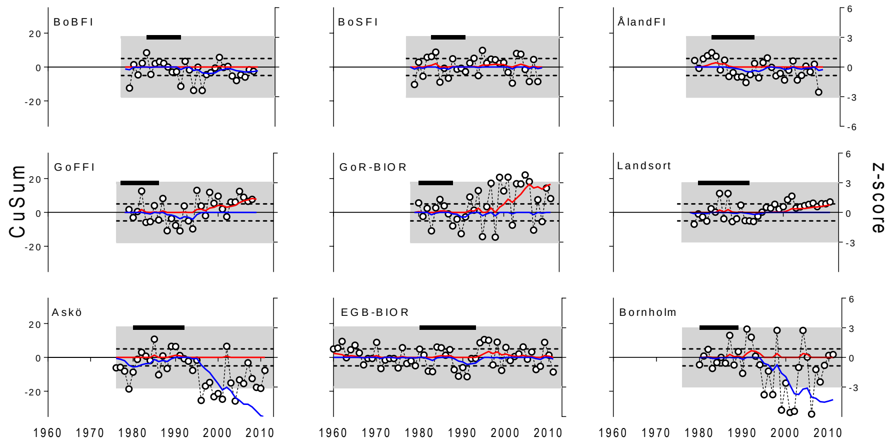

% CB, RefCon$_{Chl}$

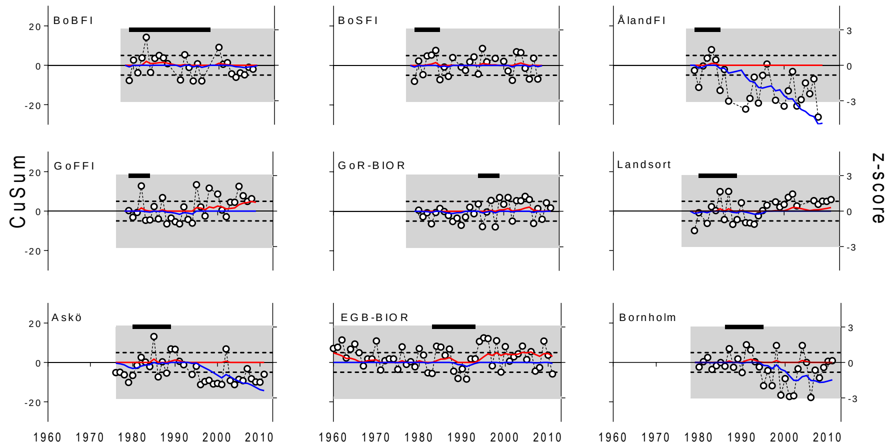

Figure S3, E

MMB, Entire dataset period

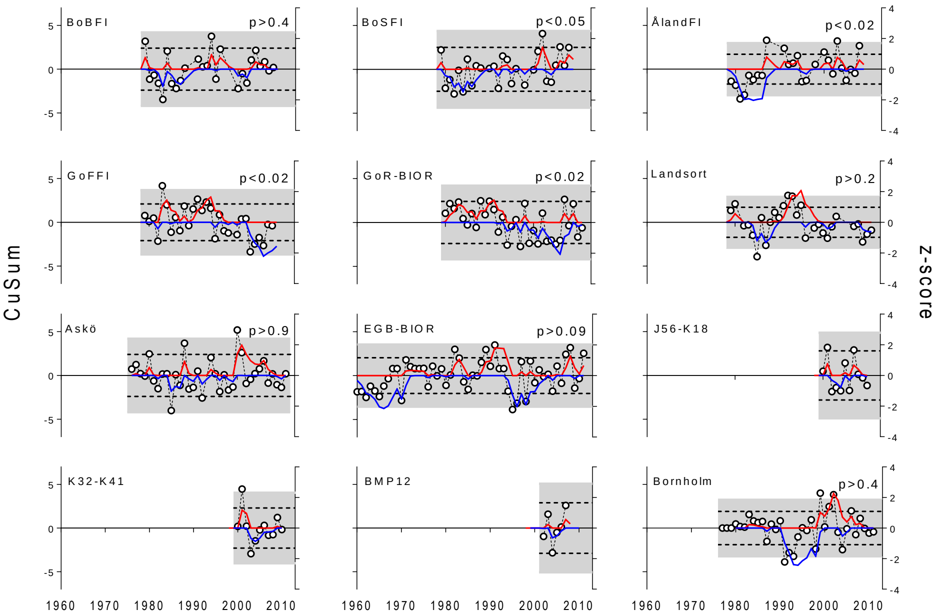

MMB, RefCon$_{Fish}$

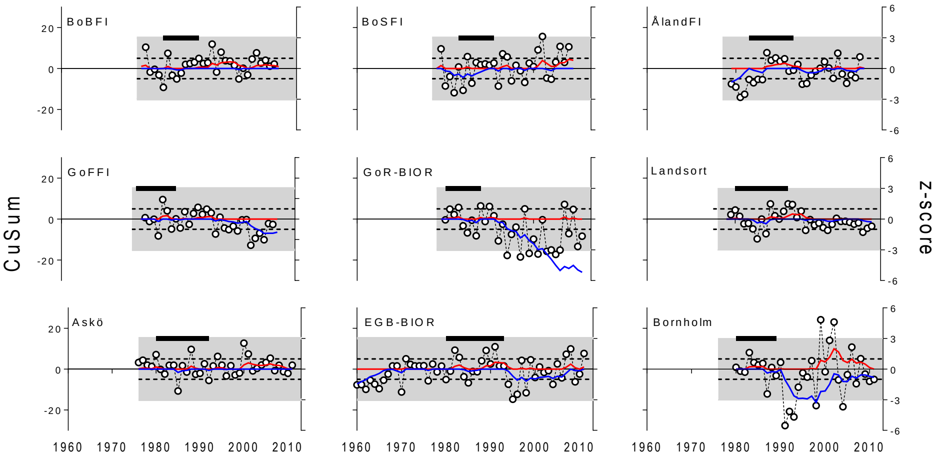

MMB, RefCon$_{Chl}$

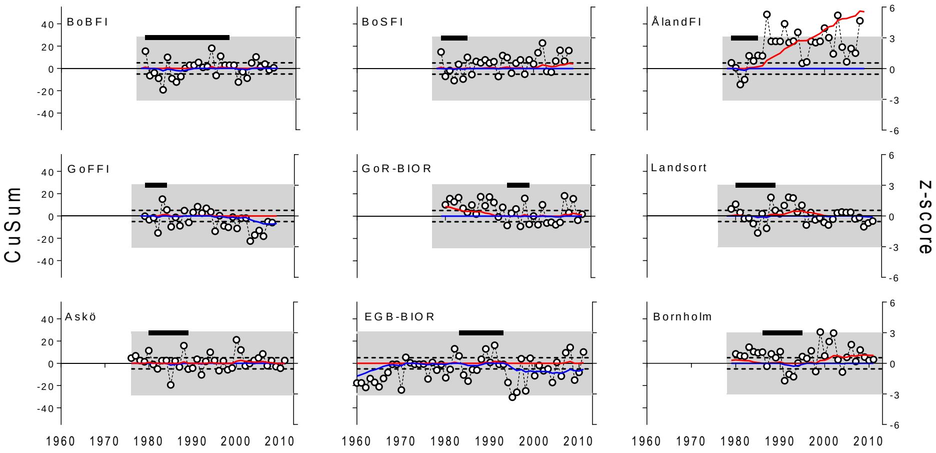

Figure S3, F

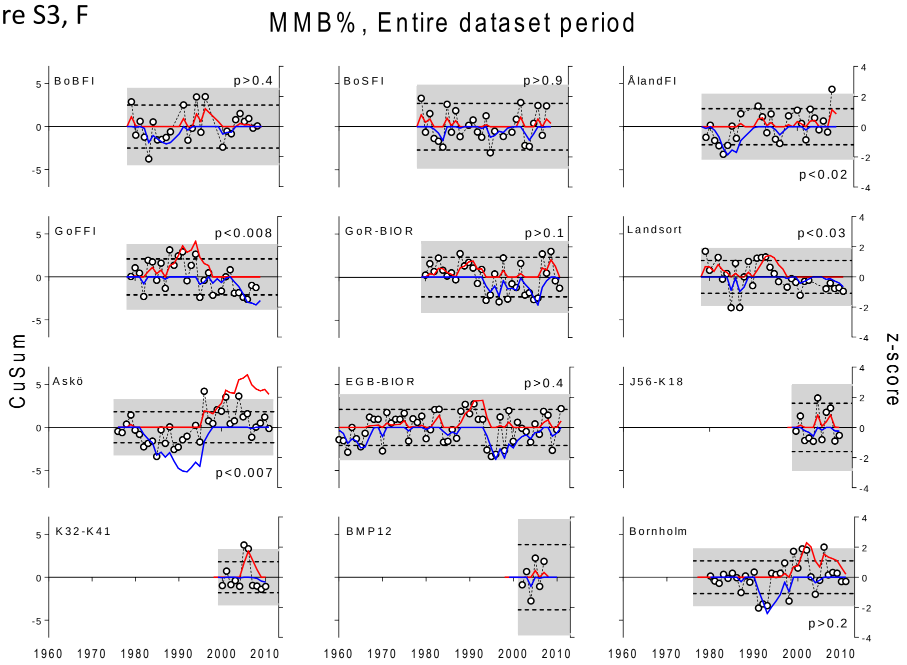
MMB%, Entire dataset period
BoBFI p>0.4
BoSFI p>0.9
ÅlandFI p<0.02
GoFFI p<0.008
GoR-BIOR p>0.1
Landsort p<0.03
Askö p<0.007
EGB-BIOR p>0.4
J56-K18
K32-K41
BMP12
Bornholm p>0.2
CuSum
z-score
1960 1970 1980 1990 2000 2010

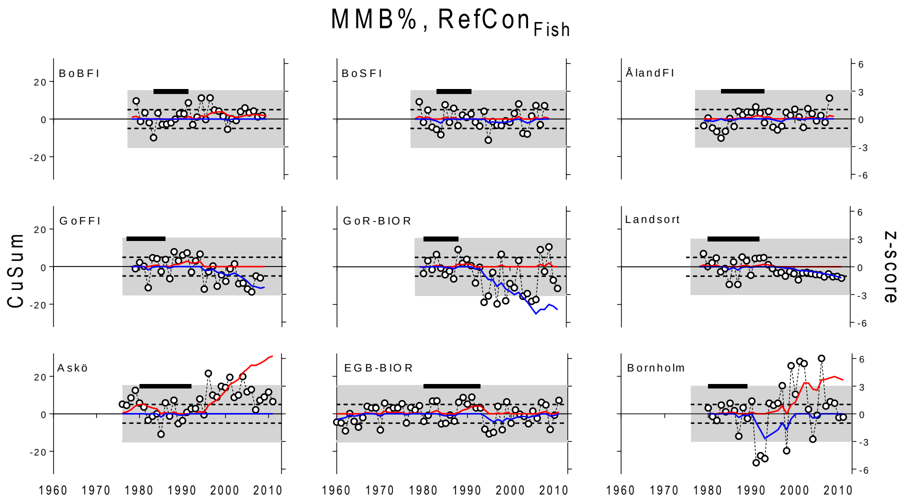
MMB%, RefCon Fish
BoBFI
BoSFI
ÅlandFI
GoFFI
GoR-BIOR
Landsort
Askö
EGB-BIOR
Bornholm
CuSum
z-score
1960 1970 1980 1990 2000 2010

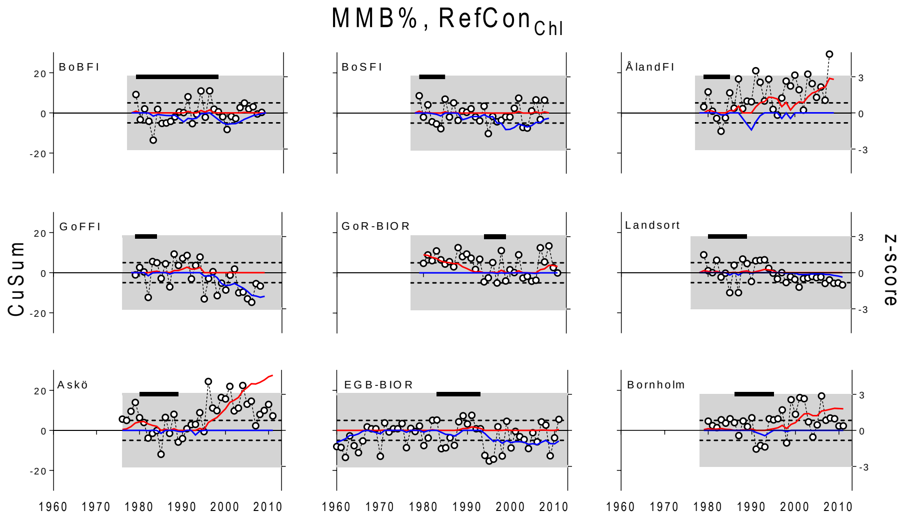
MMB%, RefCon Chl
BoBFI
BoSFI
ÅlandFI
GoFFI
GoR-BIOR
Landsort
Askö
EGB-BIOR
Bornholm
CuSum
z-score
1960 1970 1980 1990 2000 2010

Figure S3, G

RotCla/Cop, Entire dataset period

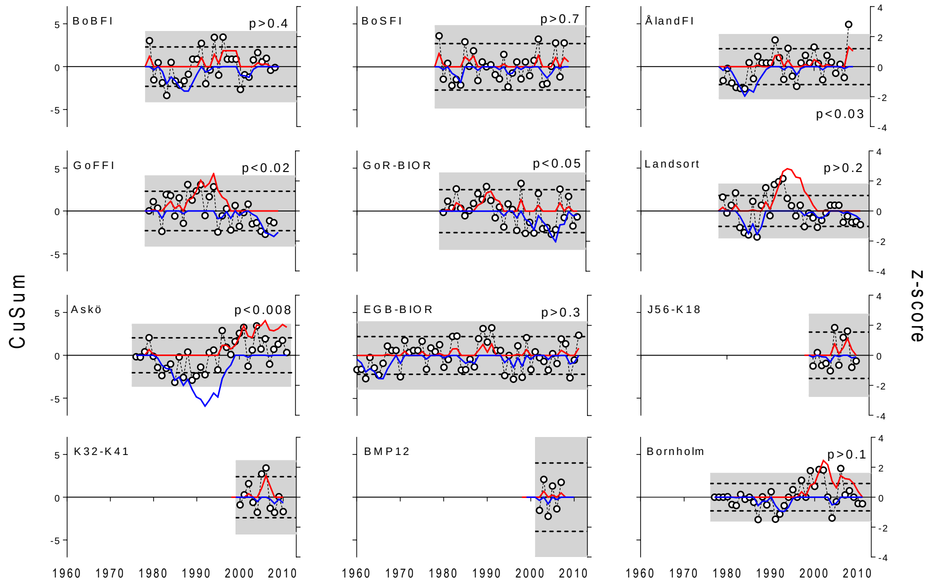

RotCla/Cop, $RefCon_{Fish}$

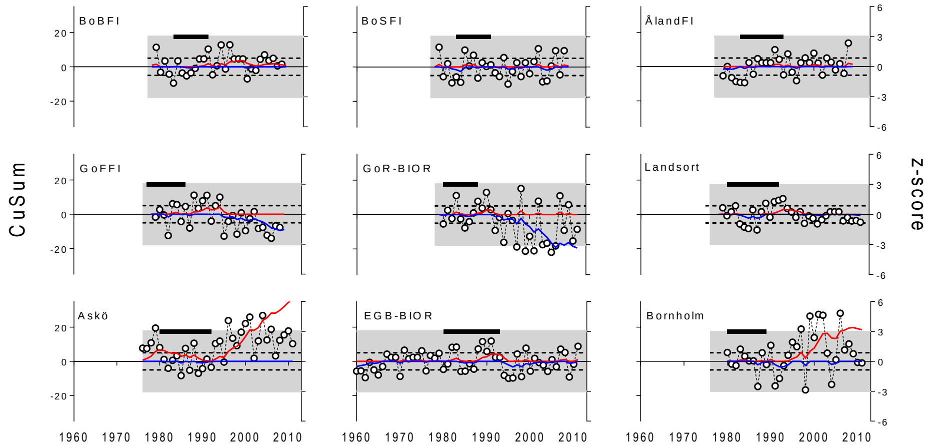

RotCla/Cop, $RefCon_{Chl}$

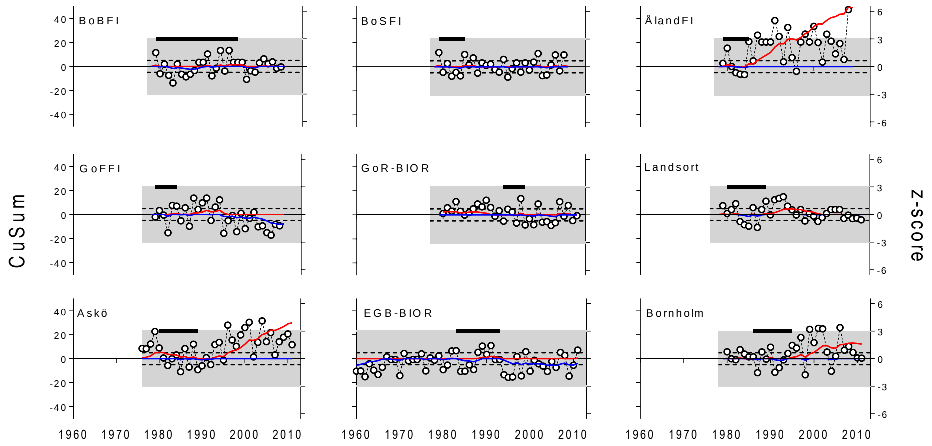

Figure S3, H

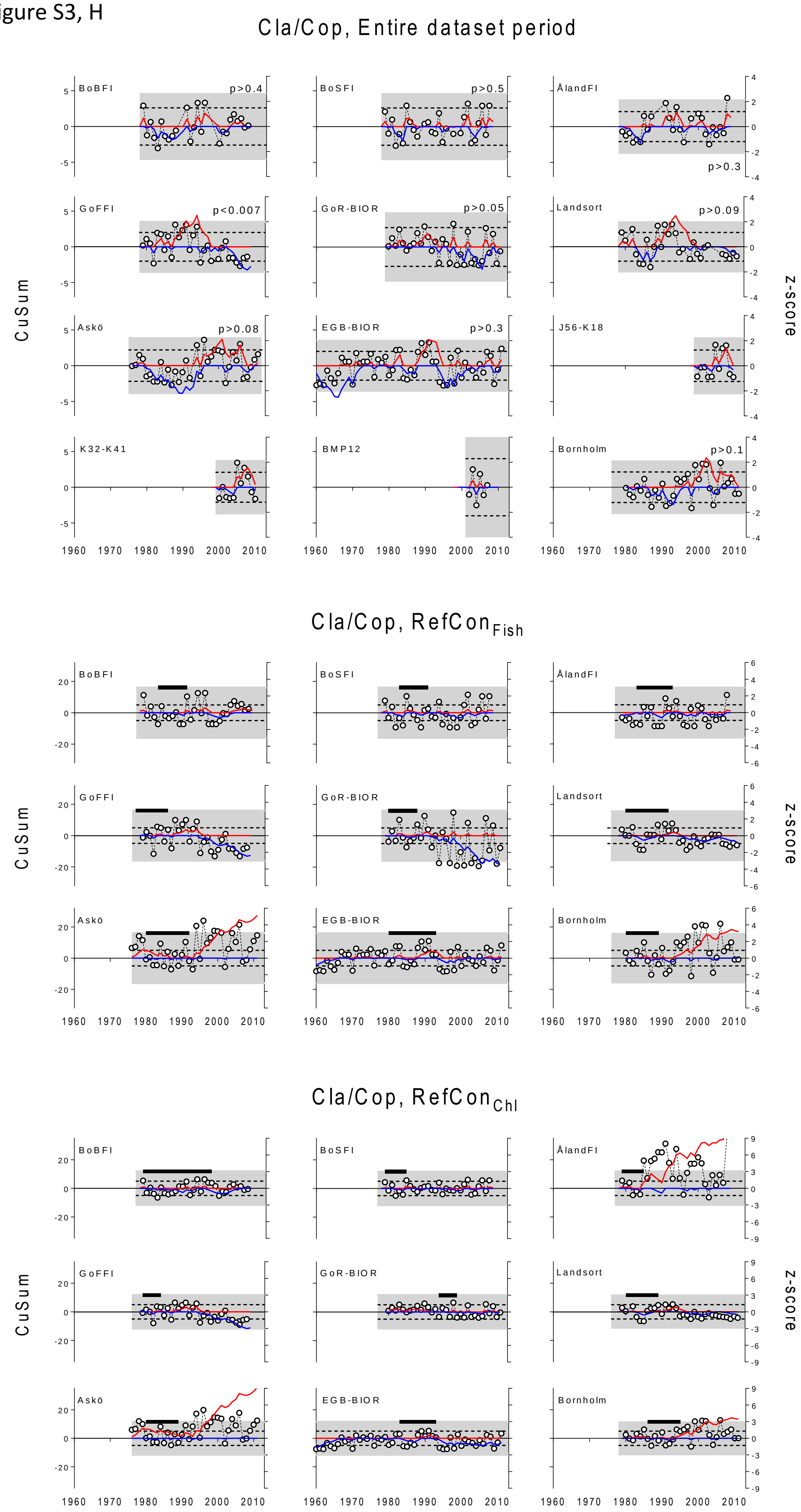

Cla/Cop, Entire dataset period
BoBFI
p>0.4
BoSFI
p>0.5
ÅlandFI
p>0.3
GoFFI
p<0.007
GoR-BIOR
p>0.05
Landsort
p>0.09
Askö
p>0.08
EGB-BIOR
p>0.3
J56-K18
K32-K41
BMP12
Bornholm
p>0.1
CuSum
z-score
1960 1970 1980 1990 2000 2010
Cla/Cop, RefCon$_{Fish}$
BoBFI
BoSFI
ÅlandFI
GoFFI
GoR-BIOR
Landsort
Askö
EGB-BIOR
Bornholm
CuSum
z-score
1960 1970 1980 1990 2000 2010
Cla/Cop, RefCon$_{Chl}$
BoBFI
BoSFI
ÅlandFI
GoFFI
GoR-BIOR
Landsort
Askö
EGB-BIOR
Bornholm
CuSum
z-score
1960 1970 1980 1990 2000 2010

Figure S3, I

MeanSize, Entire dataset period

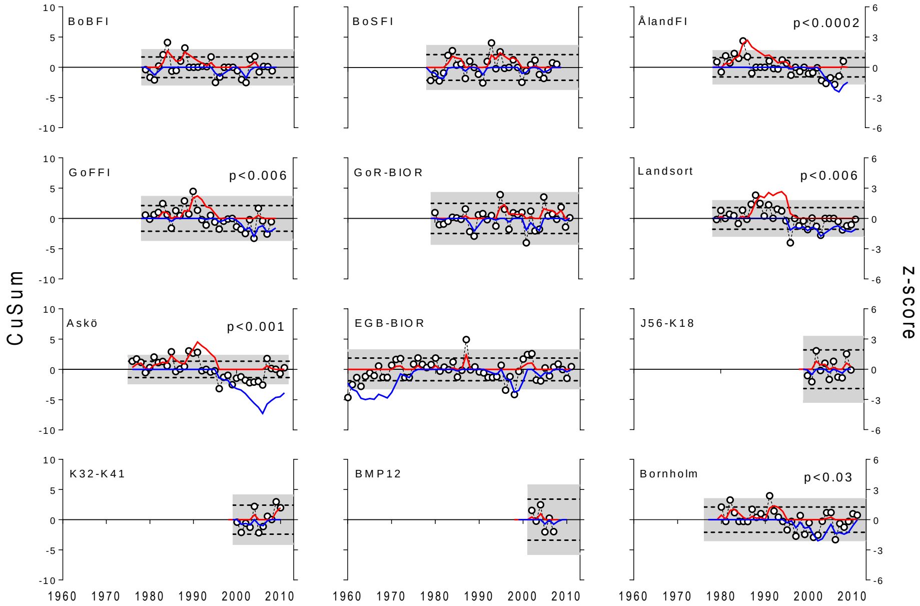

MeanSize, $RefCon_{Fish}$

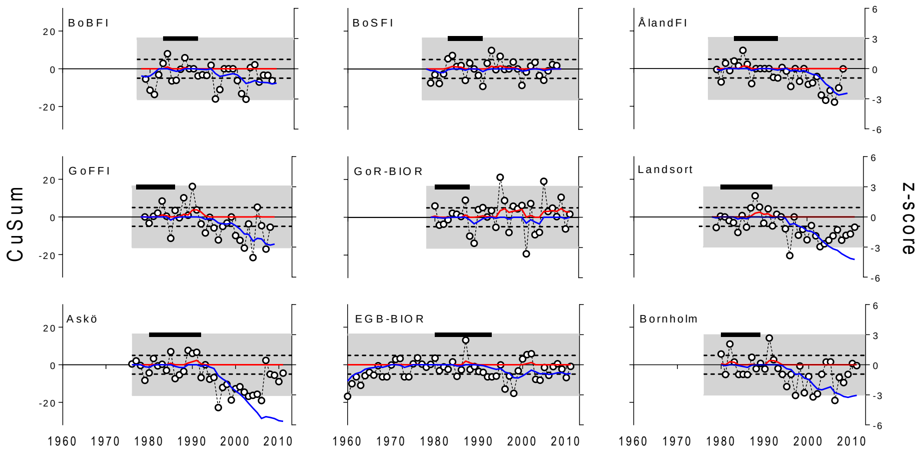

MeanSize, $RefCon_{Chl}$

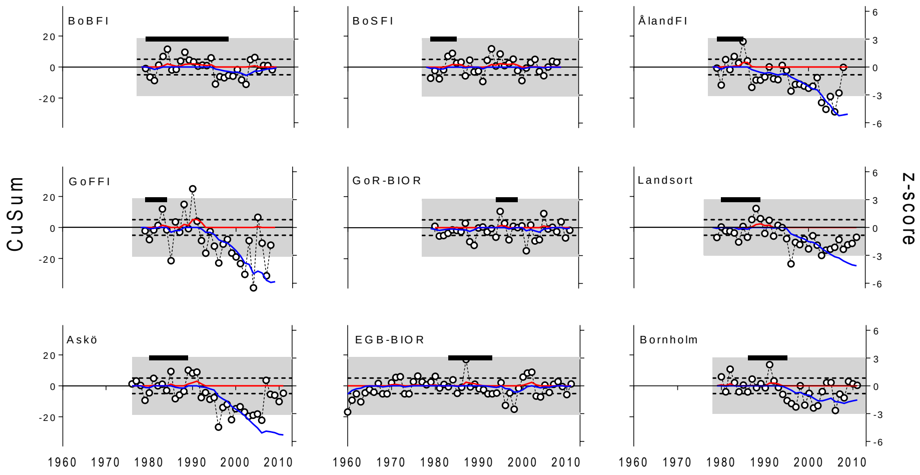

Supplement: S3 Fig — Upper (red line) and lower (blue line) DI-CuSums and Shewhart z-scores (open circles) are shown on the left and right y-axes, respectively. A shaded area represents in-control Shewhart limits and dashed lines represent upper (UCL) and lower (LCL) CuSum limits. The upper and lower control limits, were defined as either 99%-CIs around the mean values (for baseline based on an entire dataset) or using a conservative approach of ±3σ and ±5σ for Shewhart and CuSum control limits, respectively (for baseline based on either RefConFish or RefConChl). The p-values indicate significance for the non-parametric Mann—Kendall (Kendall, 1975) test for a monotonic downward or upward trend. (A) TZA, (B) TZB, (C) CB, (D) CB%, (E) MMB, (F) MMB%, (G) RotCla/Cop, (H) Cla/Cop, and (I) MeanSize. See Table 1 for details on the data origin, Table 2 for the indicator description, and Table 3 for the synthesis of the violations presented in S3 Fig. (PDF) [file pone.0158326.s003.pdf]
