# Supplementary material for: Indicator Properties of Baltic Zooplankton for Classification of Environmental Status within Marine Strategy Framework Directive
Source: PLoS One. 2016 Jul 13;11(7):e0158326. doi: 10.1371/journal.pone.0158326 (PMC4943737; doi:10.1371/journal.pone.0158326)
Supplement: S4 Fig — (PDF) [file pone.0158326.s004.pdf]

PLoS One | Supporting Information

Indicator properties of Baltic zooplankton for classification of environmental status within Marine Strategy Framework Directive

Elena Gorokhova[1*], Maiju Lehtiniemi[2], Lutz Postel[3], Gunta Rubene[4], Callis Amid[1], Jurate Lesutiene[5], Laura Uusitalo[2], Solvita Strake[6] and Natalja Demereckiene[7]

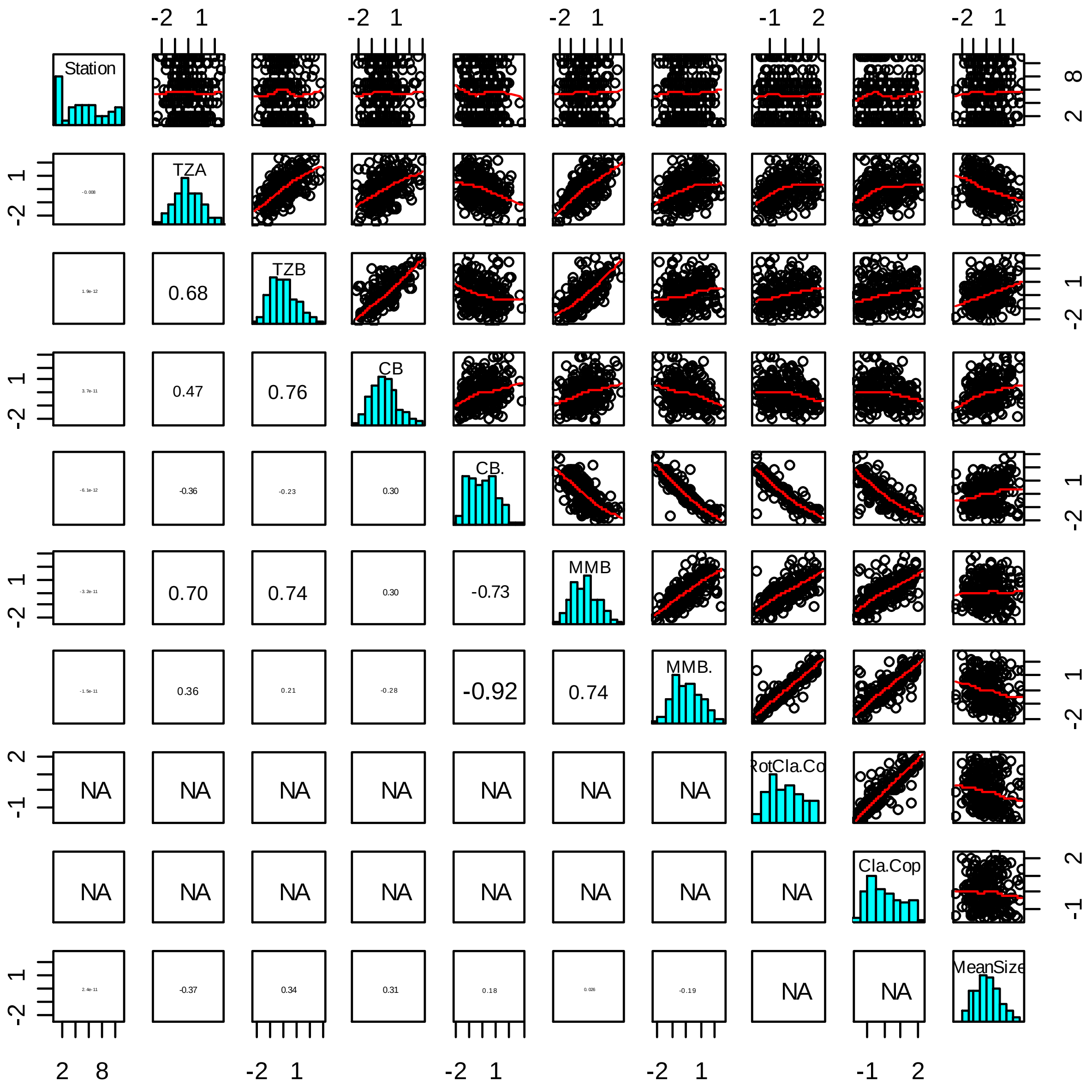

S4 Fig. Pairplot showing correlations between the indicators for all datasets combined.
