# Supplementary material for: Indicator Properties of Baltic Zooplankton for Classification of Environmental Status within Marine Strategy Framework Directive
Source: PLoS One. 2016 Jul 13;11(7):e0158326. doi: 10.1371/journal.pone.0158326 (PMC4943737; doi:10.1371/journal.pone.0158326)
Supplement: S5 Fig — Significantly higher and less variable prediction accuracy was obtained for identification of zooplankton community structure as being outside of the reference values in the RefConFish models (B; Wilcoxon matched-pairs signed rank test, p < 0.004). (PDF) [file pone.0158326.s005.pdf]

PLoS One │ Supporting Information

Indicator properties of Baltic zooplankton for classification of environmental status within Marine Strategy Framework Directive

Elena Gorokhova[1*], Maiju Lehtiniemi[2], Lutz Postel[3], Gunta Rubene[4], Callis Amid[1], Jurate Lesutiene[5], Laura Uusitalo[2], Solvita Strake[6] and Natalja Demereckiene[7]

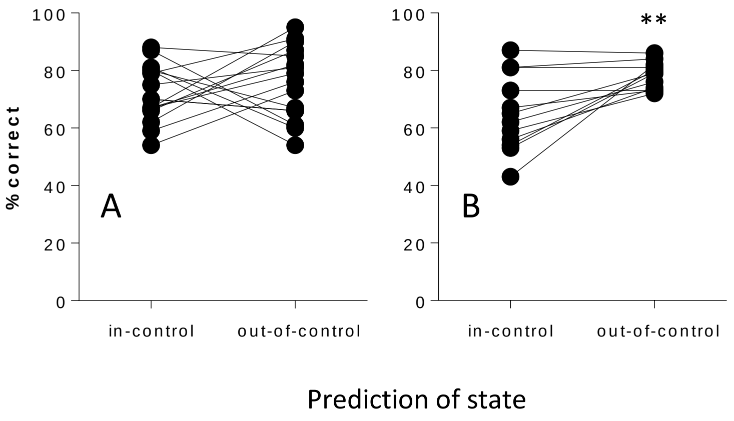

S5 Fig. Classification accuracy for binary logistic models predicting zooplankton community structure being in the reference state (in-control) or not (out-of-control) for RefCon$_{Chl}$ (A) and RefCon$_{Fish}$ (B); see Table 4 for the list of models and their specifications. Significantly higher and less variable prediction accuracy was obtained for identification of zooplankton community structure as being outside of the reference values in the RefCon$_{Fish}$ models (B; Wilcoxon matched-pairs signed rank test, $p < 0.004$).
