# Supplementary material for: Indicator Properties of Baltic Zooplankton for Classification of Environmental Status within Marine Strategy Framework Directive
Source: PLoS One. 2016 Jul 13;11(7):e0158326. doi: 10.1371/journal.pone.0158326 (PMC4943737; doi:10.1371/journal.pone.0158326)
Supplement: S1 Table — (PDF) [file pone.0158326.s006.pdf]

PLoS One │ Supporting Information

Indicator properties of Baltic zooplankton for classification of environmental status within Marine Strategy Framework Directive

Elena Gorokhova, Maiju Lehtiniemi, Lutz Postel, Gunta Rubene, Callis Amid, Jurate Lesutiene, Laura Uusitalo, Solvita Strake and Natalja Demereckiene

Zooplankton sampling

According to Helsinki Commission (HELCOM) guidelines for biological monitoring [1], zooplankton were collected by vertical tows from either ~5 m above the bottom to the surface (shallow stations, ≤ 30 m) or in depth layers (deep stations, ≥ 30 m) as specified by the regional monitoring programs. Most commonly, a WP2 net with mouth opening of 57 cm and equipped with a flow meter was used; see, however, Table 1 for details on deviations in sampling methods in different laboratories. Fully recognizing the patchiness and variability inherent in mesozooplankton sampling, we assumed that our estimates based on the relatively uniform sampling are comparable.

Sample analysis

Samples were preserved in 4% buffered formalin and analyzed within the national monitoring programs (Supporting Information, Table S1). Copepods were classified according to species, developmental stage, and sex (adults). For the stage classification, all nauplial stages were pooled into a single category and copepodites CI-III and CIV-V were classified as younger and older copepodites, respectively. Rotifers and cladocerans were identified to the lowest possible taxonomic level. In most laboratories, the latter were also classified according to sex, ovigerous or non-ovigerous females or, alternatively, as size classes. Biomass was estimated using individual wet weights and size classes [2]; for species not included in this source, either measured or calculated individual weights based on length measurements were used [3-5].

Table S1. Details for zooplankton analysis methods employed in the national laboratories.

Code for the dataset	Institute, country	Preservation	Sub-sampling equipment	Magnification, specimens counted	Biomass assessment
Askö, Landsort	Systems Ecology, Stockholm University, Sweden	Buffered (di-sodium tetraborate) formalin, 4%	Kott splitter [6]	×80, ≥500	Standard stage-and taxa-specific individual weight factors [2]
GoFFI, ÅlandFI, BoSFI, BoBFI	Finnish Institute of Marine Research/Finnish Environment Institute, Finland	Buffered (hexamine) formalin, 4%	Folsom splitter		
GoR-BIOR, EGB-BIOR	Institute of Food Safety, Animal Health and Environment, Latvia	Buffered (di-sodium tetraborate) formalin, 4%	Stempel-pipette (2 mL)	×12,6-80, ≥300	
K32/41, J56/K18, BMPJ2	Environmental Protection Agency, Marine Research Department, Lithuania	Unbuffered formalin, 4%	Plunger Sampling Pipette (0.5 mL)	×70, ≥500	
Bornholm	Leibniz Institute for Baltic Sea Research, Germany	Buffered (di-sodium tetraborate) formalin, 4%	Calibrated Eppendorf Pipette, wide mouth (0.5 mL)	×40, ≥300	Cladocerans are classified according to species only; the biomass is calculated using genus-specific individual wet mass. For other zooplankton, standard stage-and taxon-specific individual wet mass are applied [2].

References

1. HELCOM. Guidelines for the Baltic monitoring programme for the third stage. Part D. Biological determinants. Balt Sea Environ Proc. 1988; 27D: 1-161.
2. Hernroth L. Recommendations on methods for marine biological studies in the Baltic Sea. Mesozooplankton biomass assessment. BMB Publ. 1985; No. 10: 1-32.
3. Kankaala P, Johansson S. The influence of individual variation on length-biomass regressions in three crustacean zooplankton species. J Plankton Res. 1986; 8: 1027-1038.
4. Burkhardt S. Seasonal size variation in the predatory cladoceran *Bythotrephes cederstroemii* in Lake Michigan. Freshwat Biol. 1994; 31: 97-108.
5. Uitto A, Gorokhova E, Välipakka P. Distribution of the non-indigenous *Cercopagis pengoi* in the coastal waters of the eastern Gulf of Finland. ICES J Mar Sci. 1999; 56: 49-57.
6. Kott M. Modified whirling apparatus for the subsampling of plankton. Aust J Mar Freshw Res. 1953; 4:387–393.
