# Supplementary material for: Indicator Properties of Baltic Zooplankton for Classification of Environmental Status within Marine Strategy Framework Directive
Source: PLoS One. 2016 Jul 13;11(7):e0158326. doi: 10.1371/journal.pone.0158326 (PMC4943737; doi:10.1371/journal.pone.0158326)
Supplement: S2 Table — Significant correlations at p < 0.05 are in bold; n–number of samples (i.e., the number of years included in the dataset). See Tables 1 and 2 for indicator and dataset descriptions. (PDF) [file pone.0158326.s007.pdf]

Supporting Information, Table S2. Pearson *r* correlations among the indicators in each dataset. Significant correlations at $p < 0.05$ are in bold; *n* – number of samples (i.e., length of the dataset) used in the analysis. See Tables 1 and 2 for indicator and dataset descriptions.

		TZA	TZB	CB	CB%	MMB	MMB%	RotCla/Cop	Cla/Cop
BoBFI	TZB	0.37							
(*n*=25)	CB	0.16	**0.80**						
	CB%	-0.36	-0.29	0.21					
	MMB	**0.45**	**0.68**	0.17	**-0.88**				
	MMB%	0.39	0.34	-0.24	**-0.96**	**0.91**			
	RotCla/Cop	0.37	0.37	-0.21	**-0.95**	**0.91**	**0.99**		
	Cla/Cop	0.34	0.34	-0.22	**-0.96**	**0.90**	**0.99**	**0.99**	
	MeanSize	**-0.46**	**0.63**	**0.62**	-0.01	0.30	0.02	0.07	0.08
BoSFI	TZB	**0.61**							
(*n*=27)	CB	**0.52**	**0.95**						
	CB%	-0.20	0.16	**0.44**					
	MMB	**0.64**	**0.55**	0.29	**-0.71**				
	MMB%	0.23	-0.13	-0.41	**-0.99**	**0.74**			
	RotCla/Cop	0.23	-0.04	-0.34	**-0.97**	**0.78**	**0.97**		
	Cla/Cop	0.24	0.02	-0.25	**-0.93**	**0.78**	**0.92**	**0.94**	
	MeanSize	-0.34	**0.50**	**0.57**	**0.50**	-0.11	-0.49	-0.37	-0.30
ÅlandFI	TZB	**0.75**							
(*n*=25)	CB	**0.69**	**0.96**						
	CB%	-0.31	-0.07	0.16					
	MMB	**0.69**	**0.64**	**0.45**	**-0.79**				
	MMB%	0.28	0.09	-0.15	**-0.98**	**0.80**			
	RotCla/Cop	0.20	0.12	-0.12	**-0.95**	**0.79**	**0.97**		
	Cla/Cop	-0.05	0.06	-0.12	**-0.75**	**0.61**	**0.77**	**0.85**	
	MeanSize	**-0.65**	-0.02	0.02	0.32	-0.26	-0.28	-0.13	0.18

Table c. Continued.

		TZA	TZB	CB	CB%	MMB	MMB%	RotCla/Cop	Cla/Cop
GoFFI	TZB	**0.79**							
(*n*=29)	CB	**0.80**	**0.82**						
	CB%	-0.26	**-0.45**	0.05					
	MMB	**0.67**	**0.91**	**0.54**	**-0.76**				
	MMB%	0.27	**0.54**	0.01	**-0.95**	**0.83**			
	RotCla/Cop	0.26	**0.53**	-0.01	**-0.95**	**0.82**	**0.99**		
	Cla/Cop	0.25	**0.54**	0.01	**-0.94**	**0.83**	**0.99**	**0.99**	
	MeanSize	-0.28	0.31	0.04	-0.30	0.39	**0.46**	**0.45**	**0.47**
Landsort	TZB	**0.72**							
(*n*=31)	CB	**0.47**	**0.47**						
	CB%	-0.12	-0.32	**0.62**					
	MMB	**0.70**	**0.74**	0.01	**-0.67**				
	MMB%	0.20	0.26	**-0.60**	**-0.92**	**0.76**			
	RotCla/Cop	0.27	0.42	**-0.43**	**-0.86**	**0.75**	**0.87**		
	Cla/Cop	0.20	0.38	**-0.43**	**-0.79**	**0.74**	**0.85**	**0.91**	
	MeanSize	-0.18	0.52	0.07	-0.33	0.23	0.16	0.29	0.31
Askö	TZB	**0.53**							
(*n*=36)	CB	0.33	**0.93**						
	CB%	-0.29	0.29	**0.55**					
	MMB	**0.75**	**0.52**	0.25	**-0.45**				
	MMB%	0.23	**-0.48**	**-0.70**	**-0.81**	0.34			
	RotCla/Cop	0.28	-0.36	**-0.63**	**-0.88**	**0.47**	**0.94**		
	Cla/Cop	0.26	-0.28	**-0.48**	**-0.71**	0.36	**0.76**	**0.80**	
	MeanSize	-0.40	**0.48**	**0.66**	**0.69**	-0.19	**-0.81**	**-0.72**	**-0.57**

Table c. Continued.

		TZA	TZB	CB	CB%	MMB	MMB%	RotCla/Cop	Cla/Cop
GoR-BIOR	TZB	**0.91**							
(*n*=32)	CB	**0.61**	**0.60**						
	CB%	**-0.76**	**-0.87**	-0.17					
	MMB	**0.92**	**0.96**	**0.55**	**-0.87**				
	MMB%	**0.83**	**0.81**	0.40	**-0.79**	**0.93**			
	RotCla/Cop	**0.82**	**0.90**	0.26	**-0.97**	**0.93**	**0.88**		
	Cla/Cop	**0.81**	**0.91**	0.31	**-0.96**	**0.93**	**0.87**	**0.98**	
	MeanSize	-0.14	0.25	0.10	-0.28	0.15	0.02	0.22	0.29
EGB-BIOR	TZB	**0.95**							
(*n*=52)	CB	**0.51**	**0.54**						
	CB%	**-0.62**	**-0.65**	0.26					
	MMB	**0.82**	**0.85**	0.08	**-0.93**				
	MMB%	**0.62**	**0.64**	-0.22	**-0.98**	**0.93**			
	RotCla/Cop	**0.59**	**0.62**	-0.25	**-0.96**	**0.89**	**0.95**		
	Cla/Cop	**0.65**	**0.69**	-0.18	**-0.98**	**0.94**	**0.98**	**0.96**	
	MeanSize	0.13	**0.42**	**0.28**	-0.23	**0.31**	0.20	0.24	0.26
J56-K18	TZB	**0.70**							
(*n*=11)	CB	0.47	**0.91**						
	CB%	**-0.66**	-0.06	0.17					
	MMB	**0.93**	0.53	0.27	**-0.82**				
	MMB%	0.54	0.02	-0.21	**-0.94**	**0.75**			
	RotCla/Cop	0.36	-0.07	-0.29	**-0.87**	**0.63**	**0.96**		
	Cla/Cop	0.23	-0.09	-0.30	**-0.74**	0.54	**0.87**	**0.94**	
	MeanSize	**-0.64**	0.02	0.21	**0.90**	**-0.73**	**-0.79**	**-0.68**	-0.47

Table c. Continued.

		TZA	TZB	CB	CB%	MMB	MMB%	RotCla/Cop	Cla/Cop
K32-41	TZB	**0.95**							
(*n*=11)	CB	**0.87**	**0.97**						
	CB%	0.01	0.19	0.38					
	MMB	**0.97**	**0.92**	**0.81**	-0.12				
	MMB%	0.02	-0.20	-0.39	**-0.82**	0.16			
	RotCla/Cop	0.14	-0.05	-0.22	**-0.61**	0.26	**0.83**		
	Cla/Cop	0.04	-0.15	-0.26	-0.37	0.05	0.53	0.31	
	MeanSize	-0.12	0.07	0.22	0.89	-0.19	**-0.66**	-0.56	-0.36
BMP12	TZB	**0.80**							
(*n*=6)	CB	0.39	**0.84**						
	CB%	-0.38	0.22	0.67					
	MMB	**0.92**	0.57	0.06	-0.63				
	MMB%	0.47	-0.12	-0.58	**-0.98**	0.71			
	RotCla/Cop	0.59	0.09	-0.39	**-0.87**	**0.80**	**0.90**		
	Cla/Cop	0.41	-0.03	-0.39	-0.78	0.64	**0.83**	**0.91**	
	MeanSize	-0.57	-0.01	0.46	**0.95**	-0.73	**-0.96**	**-0.85**	-0.74
Bornholm	TZB	**0.84**							
(*n*=32)	CB	0.29	**0.70**						
	CB%	**-0.56**	-0.09	**0.54**					
	MMB	**0.70**	**0.39**	-0.21	**-0.83**				
	MMB%	**0.51**	0.09	**-0.51**	**-0.92**	**0.91**			
	RotCla/Cop	**0.58**	0.13	**-0.50**	**-0.95**	**0.82**	**0.90**		
	Cla/Cop	**0.53**	0.08	**-0.54**	**-0.95**	**0.80**	**0.93**	**0.92**	
	MeanSize	**-0.49**	0.01	**0.59**	**0.87**	**-0.66**	**-0.79**	**-0.85**	**-0.82**
